# MXene-based photocatalysts for organic pollutant degradation: synthesis, functional properties, mechanisms, and kinetic insights

**DOI:** 10.1039/d6ra08566k

**Published:** 2026-10-15

**Authors:** Naveed Ahmad, Sheraz Ahmad, Salman Khan, Nolubabalo Matinise, Abdulkarim Alansari

**Affiliations:** a Department of Chemical and Materials Engineering, College of Engineering, Northern Border University Arar Saudi Arabia; b Department of Chemistry, Abdul Wali Khan University Mardan Mardan 23200 Pakistan; c UNESCO-UNISA Africa Chair in Nanosciences/Nanotechnology, College of Graduate Studies, University of South Africa Pretoria 0181 South Africa matinn@unisa.ac.za; d Mechanical Engineering Department, College of Engineering, Northern Border University Arar Saudi Arabia

## Abstract

The rapid proliferation of organic pollutants in aquatic environments has intensified the search for efficient and scalable remediation technologies. MXene-based materials, a growing class of two-dimensional transition metal carbides, nitrides, and carbonitrides, have emerged as highly promising photocatalysts owing to their exceptional electrical conductivity, tunable surface chemistry, high surface area, and broad light absorption. This review comprehensively examines the engineering of MXene-based composites for the photocatalytic degradation of organic pollutants, covering key synthesis strategies, including hydrothermal, solvothermal, *in situ*, self-assembly, and calcination routes. The structural diversity of MXene families and the physicochemical properties governing their photocatalytic performance, including synergistic composite interactions, photocatalytic properties, optical behavior, and biosafety, are critically discussed. Factors influencing degradation efficiency, including photocatalyst dosage, pollutant concentration, pH, and temperature, are systematically analyzed. The mechanistic pathways of pollutant mineralization and the kinetic behavior of MXene-based systems, both in the presence and absence of oxidants, are evaluated with reference to current literature. Unlike prior reviews that discuss MXenes in broader contexts, this work uniquely integrates mechanistic and kinetic analyses within a unified framework, providing targeted insights for the rational design of photocatalysts. This review concludes with future perspectives aimed at overcoming existing limitations and accelerating the practical deployment of MXene-based photocatalysts for water treatment applications.

## Abstract 2

MXene-based materials offer tunable properties for photocatalytic applications, including hydrogen evolution, pollutant degradation, and CO_2_ reduction, with synthesis methods influencing their performance.

## Graphical abstract

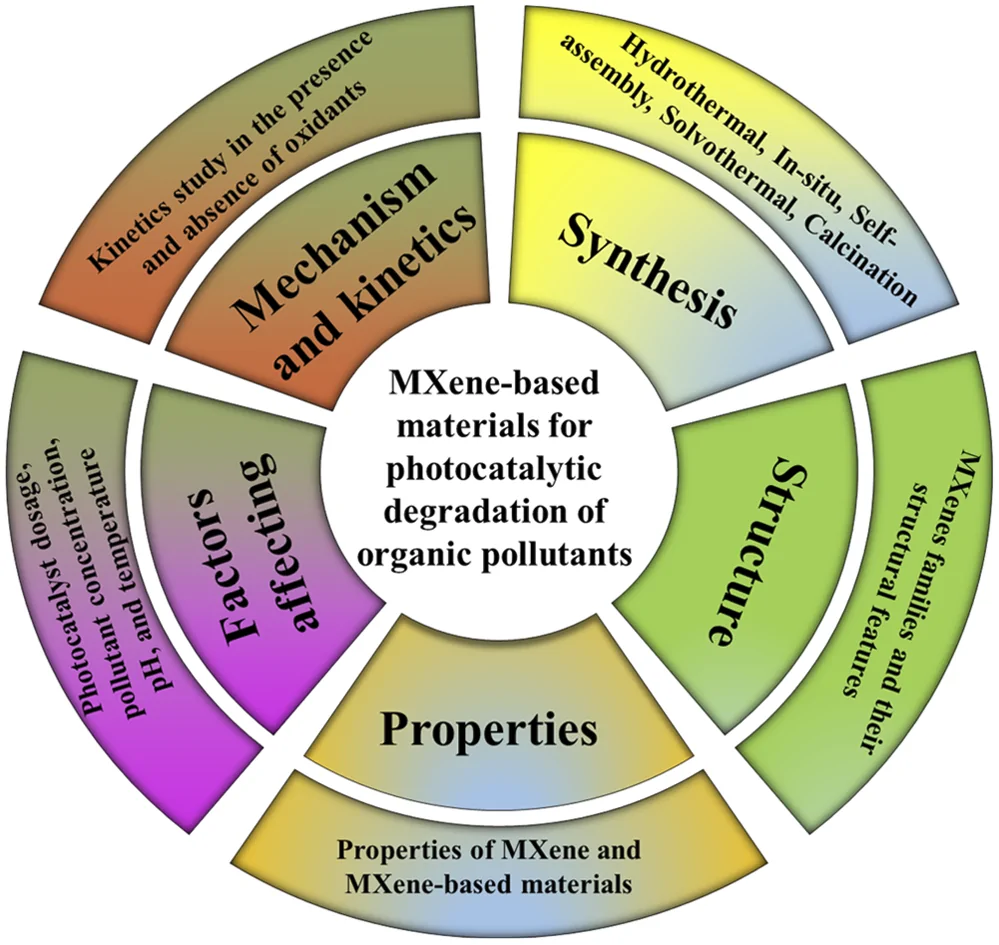

## 1. Introduction

Water is a vital component of the universe and plays a crucial role in sustaining the life of living organisms, including plants and animals. The earth's water resources consist of glaciers, oceans, rivers, lakes, mountains, and man-made water reservoirs.^1^ Water continuously moves among these resources through the hydrological cycle, which involves many natural methods such as precipitation, freezing, condensation, evaporation, and melting.^2^ Approximately 71% of terrestrial surface is covered with water. Among the total water available on earth, about 97.5% is present in oceans, whereas only 2.5% exists on land as fresh water resources.^3,4^ A major part of this land water, nearly 1.90% of the total water is reserved in ice caps and glaciers, while the remaining 0.60% is considered potable water. More than 90% of freshwater is available as groundwater under the Earth's surface. The rest of the freshwater is distributed as soil moisture, atmospheric water vapors and surface water bodies such as dams, ponds, lakes and rivers.^5^ Despite the abundance of water on earth, many global regions are facing a serious shortage of potable water. The major factors contributing to water scarcity include rapid population growth, depletion of natural water resources due to human activities, industrial development, and extensive urbanization, all of which have significantly increased water contamination.

Pollution of water bodies refers to the presence of harmful contaminants in aquatic systems at a level that makes the water unfit for cooking, bathing, drinking, and other industrial or domestic purposes.^6^ Water is considered polluted when certain substances or environmental conditions badly affect their suitability for a specific application. A wide range of pollutants can contaminate aquatic systems. Approximately, 572 emerging contaminants have been identified in the water system of Europe.^7^ Pollution generally describes the addition of harmful substances into the environment.^8^ According to the European Investment Bank, about 380 billion m^3^ of municipal wastewater is produced globally every year, and this amount is projected to rise by 24% by 2030 and by 51% by 2050.^9^ Domestic, industrial, and agricultural activities are considered the major contributors to water pollution. Industries discharge a large number of contaminants including heavy metals, toxic sludge and various hazardous chemicals into water bodies each year.^10^ Globally, about 80% of industrial waste and garbage is released directly into water resources without proper treatment. Agriculture activities consume a large amount of water and simultaneously contribute significantly to water pollution.^11^ Furthermore, a variety of hazardous contaminants, including microplastics, heavy metals, radionuclides, oils, pharmaceuticals, dyes, pesticides, and phosphorus and nitrogen containing compounds are released into the aquatic environment from different sources on regular bases. These contaminants have serious harmful effects on both aquatic and terrestrial life systems.^7^ Even at low concentrations, these pollutants show high toxicity and possess carcinogenic, bio-accumulative and bio-refractory characteristics. Among the various pollutants present in aquatic systems, organic pollutants are the most frequently observed in water bodies. Generally, these organic pollutants cause serious risks to human health as well as to an ecosystem, and most of them have been identified as mutagenic and carcinogenic in nature.^12^ Therefore, the development of highly efficient technologies for the removal of these hazardous substances from water resources has become an essential requirement.^7,13^

Among organic pollutants, pharmaceutical and personal care products (PPCPs) are considered the most frequently identified emerging pollutants in water bodies. These substances represent a major class of organic contaminants that can cause serious risks to both aquatic organisms and human health. Pharmaceuticals are complex chemical substances widely used for the treatment and prevention of different diseases in animals and humans.^14,15^ Due to their specialized designed nature, a significant portion of used pharmaceuticals is excreted either in un-metabolized form or as metabolites through urine and feces, eventually entering the public sewage systems. In addition, the improper disposal of expired pharmaceutical products directly into water bodies increases water pollution. Wastewater released from pharmaceutical manufacturing industries also represents a main source of pharmaceutical pollution in marine environments. The existence of pharmaceutical compounds in water systems has bad effects on both humans and ecosystems. Recent studies have reported the identification of pharmaceuticals remaining in surface water, groundwater, drinking water and municipal wastewater. Typically, the pharmaceuticals level in wastewater ranges from 1–600 µg L^−1^. Likewise, acidic pharmaceuticals, especially non-steroidal anti-inflammatory drugs (NSAIDs), were found in the treated water of wastewater treatment plants (WWTPs) at concentrations within the range of 10–70 µg L^−1^. Their concentrations in surface water have also been reported to be between 0–30 µg L^−1^ in various regions of Europe, North America, and Asia.^16^ The pharmaceutical industry has shown remarkable growth and has become one of the fastest growing and most influential industries worldwide. For instance, the annual consumption of ibuprofen between 2001 and 2003 was reported to be 128 tons in Germany, 180 tons in Canada, and 276 tons in Spain. During the same period the combined annual consumption of amoxicillin in Germany and Italy reached about 110 tons. In the United States, the yearly use of antibiotics was estimated to be around 23 000 tons.^17,18^ In Poland, 31.25, 4057, 551.96, and 233.63 µg L^−1^ of concentrations of ibuprofen, naproxen, diclofenac (DCF), and ketoprofen were found in wastewater, respectively. Furthermore, the ecotoxicological effects of numerous pharmaceutical compounds have been extensively reported. For instance, acetaminophen has been associated with genotoxic and hepatotoxic effects and has been also reported to reduce the reproductive ability of daphnia magna. Carbamazephine has been shown to inhibit luminescence in V. fischeri and decrease the population of non-biting midge chironomus riparius. Therefore, the removal of these contaminants from aquatic environments is necessary to prevent more ecological damage.^16^

Similarly, pesticides are another major class of organic contaminants. Various types of organic pesticides have been extensively used for many years to protect crops and improve agricultural productivity. Although pesticides provide significant benefits in crop preservation, their use in large scale can badly affect the ecosystem and may lead to a decline in biodiversity. Pesticides also cause serious threat to human health, aquatic organisms, animals, and plant species.^7^ Research studies have shown that pesticides concentrations in aquatic environments vary considerably depending on the type of water source. The different reported concentrations range from 7–121 222 ng L^−1^ in rivers and lakes, from 20–1060 ng L^−1^ in groundwater, from 23–3172 ng L^−1^ in wastewater streams, and from 141–14 629 ng L^−1^ in drinking water sources.^7,19^ Among the commonly identified pesticides, paraquat, atrazine, naphthalene, and chlorpyrifosare the more abundant.^20^ In water bodies atrazine level may exceeds 20 mg L^−1^, while a recent study reported an atrazine concentration of 0.17 µg L^−1^ in a lake in the United States.^21^ Different studies have indicated that organophosphorus pesticides are responsible for more than 100 000 deaths and approximately 2.0 million hospitalizations annually, with most cases occurring in low- and middle-income countries. In general, pesticides poisoning causes the deaths of over 150 000 people globally each year.^7,16^

Similarly, dyes are among the most common industrial contaminants directly released into water bodies.^22^ Industries such as food, textiles and pharmaceuticals extensively use dyes as coloring agents. The release of these colored compounds into water systems causes serious risks to both human health and the environment due to the contamination of water resources.^23^ Dyes are chemical substances used to give color to different materials. They are widely applied in the coloring of fibers, fabrics, paper, hair, wax, cosmetics, fur, leather and food products.^24^ Globally, about 7 × 10^5^ tons of dyes are produced annually, with more than 100 000 commercially available dye products.^25^ Dyes and heavy metals are considered major contaminants that strongly effect water quality. Large quantities of dyes are generated from industries including rubber, plastic, paper, leather, textile and printing industries.^26^ During, dyeing operations, about 10–15% of the total dye produced globally is lost and released into textile wastewater.^27^ The presence of dyes in wastewater is very harmful to living organisms due to their carcinogenic, toxic, and xenobiotic nature.^28^ Textile dyes and other industrial colorants are of specific environmental concern due to their toxicity and resistance to biodegradation. Besides their toxic effects, dye pollutants increase the chemical oxygen demand (COD) of water, which resultantly reduces dissolved oxygen levels required for marine life. Industrial wastewater containing synthetic pigments also restricts the penetration of sunlight into water bodies, thereby inhibiting the photosynthetic activity of marine organisms. As a result, biochemical oxygen demand (BOD) increases, and the food chain of aquatic ecosystem is badly affected. The degradation and removal of dyes from wastewater are tough because of their high chemical stability, complex aromatic ring structures, and strong water solubility. Dyes and colors significantly damage aquatic ecosystems by lowering dissolved oxygen concentrations and increasing COD values (150–30 000 mg L^−1^) and BOD values (80–6000 mg L^−1^). Certain dyes are also associated with severe health hazards. For instance, Direct Black 38 has been detected as a carcinogenic compound relate to bladder cancer, while Direct Blue 15 has been reported to cause mutagenic effects.^7^

Due to the toxic and hazardous nature of organic contaminants, proper attention must be given to contamination caused by pesticides, pharmaceuticals, and dyes to protect living things from their poisonous and undesirable side effects. Various treatment operations have been developed to reduce water contamination. These operations include flocculation, precipitation, adsorption, electrodialysis, membrane separation, coagulation, aerobic and anaerobic oxidation, and advanced oxidation processes (AOPs). However, wastewater treatment through physiochemical techniques often requires the use of various chemicals like NH_3_, NaOH, alum, ferric salts, chlorine compounds, coagulating agents, ion-exchange resins, filtration aids, and regenerating chemicals.^29^ In most cases, the main aim of these methods is to remove contaminants from water by transferring them from the aqueous phase into another phase, usually the solid phase. Nevertheless, these techniques frequently generate large amount of sludge, producing additional challenges related to its safe handling and disposal.^30^ Furthermore, these treatment processes required high energy consumption, operational costs, specialized engineering expertise, and a proper infrastructure for efficient operation.^29^ Among the various wastewater treatment techniques, AOPs, including SO_4_˙^−^ and ˙OH-based oxidation, ionization radiation, ozonation, and photocatalysis, are considered some of the most efficient methods for the removal of contaminants.^31^ Among these AOPs, photocatalytic degradation of organic pollutants has attracted significant attention due to its eco friendliness, cost-effectiveness, and high treatment efficiency.^32^ Photocatalysis is widely applied for the oxidation and decomposition of different organic contaminants using different photocatalytic materials. Photocatalyst are generally semiconductor materials have the ability to degrade organic contaminants in water under photo irradiation through the generation of reactive species.^33,34^ During the photocatalytic process a lot of different reactive oxidizing species are produced, including photogenerated e^−^–h^+^ pairs, ˙OH, and O_2_˙^−^. These reactive species play a vital role in the degradation and complete mineralization of organic contaminants.^35^

Among various two-dimensional (2D) materials, MXenes and MXene-based nanocomposites have appeared as highly favorable materials for photocatalytic applications owing to their outstanding electrical, chemical, thermal, mechanical and optical properties. MXenes are a recently developed class of 2D materials containing transition metal carbides, carbonitrides, nitrides, and oxycarbides, generally represents by the formula M_*n*+1_X_*n*_T_*x*_.^36^ In this formula, M represents early transition metal, X indicates carbon or nitrogen, while TX shows surface functional groups such as (–OH, –F, and –O). The value of *n* = 1–4 indicates the number of atomic layers in the within the unit cell (Fig. 1).^37^ MXenes are synthesized by selectively removing the A-layer from the MAX phase structure.^38^ In the MAX phases M represents transition metals such as Nb, V, Ti, or Ta, A corresponds to elements belonging to group 13th and 14th of the periodic table, and X denotes C or N.^39^Fig. 1(a) and (b) illustrates the structures of (a) MAX phase and (b) MXene.^37^ A major breakthrough in MXene preparation was achieved by Naguib and his co-workers,^40^ who successfully synthesized MXene through wet chemical etching of Ti_3_AlC_2_ using hydrofluoric acid. Since then, various types of MXenes have been successfully prepared from different MAX phase precursors.^41^ Now a days MXenes have gained considerable attention and are widely used in photocatalytic applications, especially wastewater treatment processes. The remarkable performance of MXenes in photocatalysis is mainly associated with different key factors: (i) the wet chemical etching process introduces large number of surface functional groups, which facilitate the formation of strong interfacial contact between MXenes and other semiconductor materials; (ii) the tunable surface chemistry of MXenes allows effective control over their bandgap alignment; and (iii) the conductive metal layers present in their structure provide excellent electrical conductivity and efficient electron accepting ability. The MXene based materials have been widely explored for the photocatalytic degradation of different environmental pollutants.^37^

**Fig. 1:**
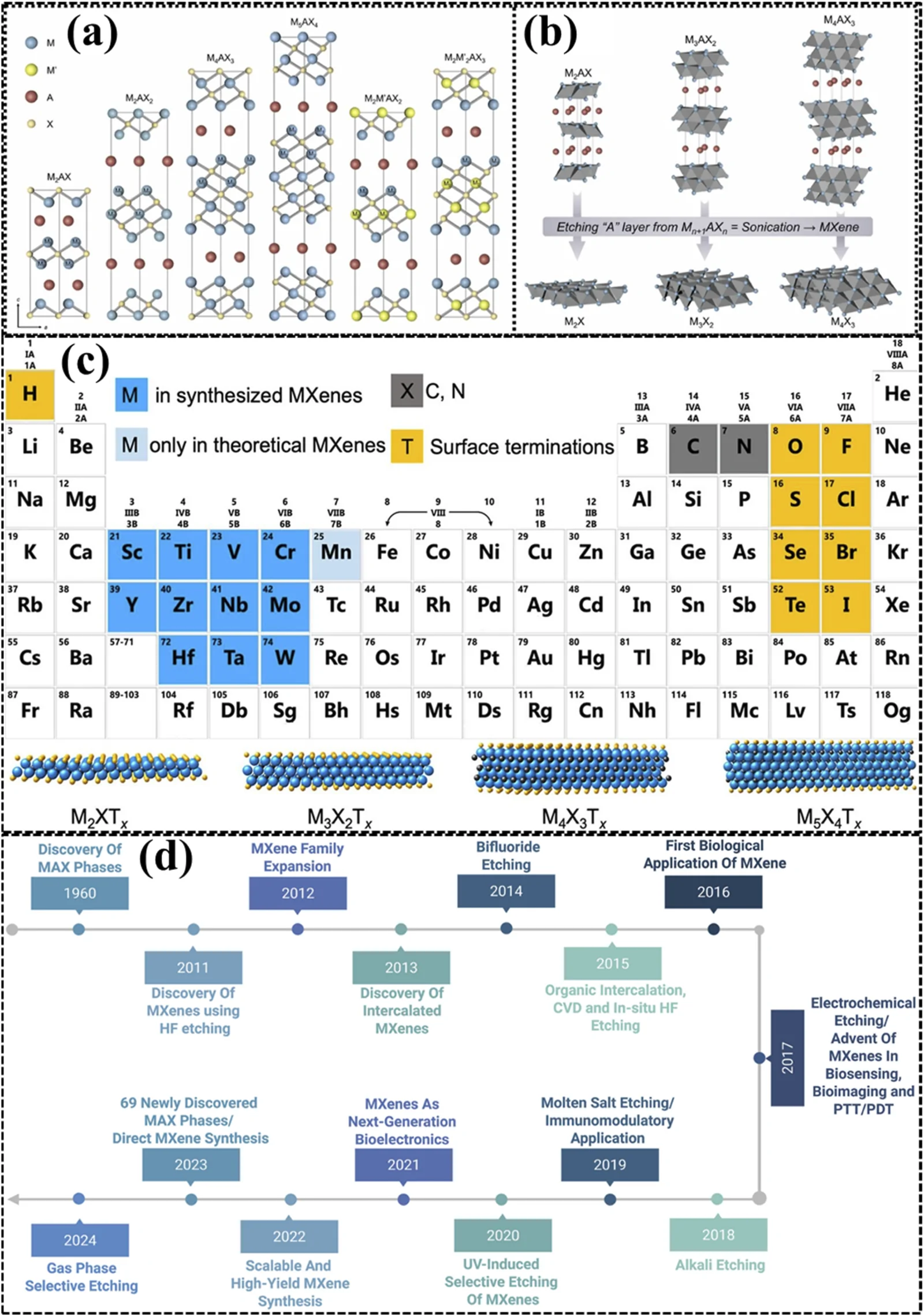
(a) Atomic structure of the MAX phase, (b) schematic representation of MXene etching. Figure reproduced with permission from ref. 42, Copyright 2022, Elsevier (c) periodic table demonstrating the chemical composition of MXenes, where the fundamental building blocks are differentiated by various colors; the bottom section presents four various MXene structure. Figure reproduced from ref. 43, which is open access and permits unrestricted use of materials under the terms of the Creative Commons CC-BY. (d) Timeline showing major milestones in MXene and MAX phase research from 1960 to 2024. Figure reproduced from ref. 44, which is open access and permits unrestricted use of materials under the terms of the Creative Commons CC-BY-NC-ND.

Among different MXenes, titanium carbide has been widely used for ecological clean up applications, especially for the photocatalytic removal of toxic pollutants. Titanium carbide possesses a flexible layered, higher surface area, outstanding electrical conductivity, and semi-metallic properties which make MXene a very promising material for photocatalytic activities.^45^ The high photocatalytic efficiency of MXene-based materials can be attributed to different properties of MXenes, including (i) efficient separation of photogenerated charge carriers (e^−^–h^+^), (ii) giving strong support for photocatalyst materials, (iii) assisting in controlling the particle size of photocatalysts, and (iv) improving the adsorption of target pollutants. The compositional diversity of MXenes across the periodic table is revealed in Fig. 1c.^43^ In addition, the distinctive surface chemistry of MXenes, especially their surface functional groups (–OH, –O, and –F) and intrinsic hydrophilicity, plays a crucial role in regulating electron (e^−^) transfer kinetics.^46,47^ The hydrophilic nature of MXenes enables superior dispersion in aqueous media and increases the interaction among the electrolyte and electrode, thereby providing efficient ion transport at the interface in electrocatalytic processes.^48^ For instance, –O terminations are known to enhance electrical conductivity and enhance charge delocalization, which activate electron transfer processes.^37^ Compared with traditional materials, MXene-based materials show superior biodegradability, biocompatibility, favorable surface characteristics, excellent electrical conductivity, and increased photocatalytic activity. Fig. 1d illustrates timeline summarizes key milestones in MXene and MAX phase research, highlighting major developments across the years 1960 to 2024.^44^

Despite the rapid development of MXene-based photocatalysts, the available literature remains dispersed across material synthesis, structural modification, photocatalytic mechanisms, and pollutant-removal applications, while a clear connection between these aspects is still lacking. Therefore, this review provides a systematic assessment of MXene-based photocatalysts, beginning with their synthesis strategies and structural characteristics and progressing toward their physicochemical and photocatalytic properties. Particular emphasis is placed on the influence of MXene structure, surface chemistry, heterostructure formation, and charge-transfer processes on photocatalytic performance, followed by a critical discussion of degradation mechanisms, kinetic behavior, and key operational parameters. The review further identifies current limitations, including oxidation stability, scalability, and practical applicability, and outlines future research directions for the rational development of efficient MXene-based photocatalysts.

## 2. Synthesis methods of MXene-derived photocatalyst

MXene possesses remarkable properties and a wide range of applications in various fields; however, their tendency restacking and limited resistance to oxidation restrict their large-scale applications.^49^ To address these limitations, the integration of MXenes with other materials is considered an effective approach. Moreover, MXene-based composite formation enhances light absorption, while reducing particle size, leading to increased surface area and improving photocatalytic performance. Various synthesis approaches, including hydrothermal, *in situ* synthesis, self-assembly, solvothermal, and calcination methods are extensively employed for the synthesis of MXene-based nanocomposites. Table 1 illustrates different synthesis approaches, key features, applications, advantages, and shortcomings of MXene-based materials.

**Table 1:** Summary of different preparation approaches of MXene-based materials^50^

Synthesis approach	Key features	Applications	Advantages	Shortcomings
Hydrothermal	High temperature and pressure, aqueous-based	Photocatalysis, adsorption	Good morphology control, high crystallinity, simple	Energy-intensive, long reaction time
*In situ*	Functional materials grow in the presence of MXene-based materials	Environmental remediation, energy storage, catalysis, and sensing	Strong interfacial interaction, uniform distribution, simple synthesis procedure	Requires precise control of synthesis conditions
Electrostatic self-assembly	Surface charge-driven assembly	Photocatalysis, electronics	Mild conditions, good dispersion	Low stability under harsh conditions
Solvothermal	Organic solvent-based, high pressure and temperature	Catalysis, sensors	Wide range of morphologies, high product yield	Costly solvents, solvent toxicity
Calcination	Thermal treatment process of MXene-based photocatalyst at elevated temperature	Photocatalysis and energy storage	Enhances crystallinity and stability	Can cause oxidation or layer restacking

### 2.1 Hydrothermal method

Hydrothermal synthesis is a process in which materials crystalize under elevated temperature and high pressures.^51,52^ This process is performed in a closed system using water as a reaction medium. If water is replaced by another liquid, the process is referred to as solvothermal.^53^ It is an effective synthesis approach that provides good control over particle shape due to its low-temperature operation and ease of process control.^54^ Hydrothermal synthesis offers good control over particle morphology and crystallinity under relatively mild reaction conditions; however, the product yield and scalability depend on the specific precursor system and reaction parameters. Reported studies have demonstrated high yields and gram-scale production under optimized conditions, highlighting the potential of this approach for scalable nanomaterial synthesis.^55,56^

Different researchers used this method for the synthesis of MXene-based materials. For example, Guo *et al.*^57^ employed a hydrothermal approach to synthesize MXene–MoS_2_ composites; whereas, pristine MXene was synthesized through HF etching process (Fig. 2a). For the synthesis of MXene–MoS_2_ composites, MAX phase, sodium molybdate (Na_2_MoO_4_), thiourea (CH_4_N_2_S), and malonic acid (C_3_H_4_O_4_) were used as precursor materials. Fig. 2b–e shows the scanning electron microscopy (SEM) micrographs of the as-prepared composite. Similarly, Gao *et al.*^58^ synthesized Fe_3_O_4_–MXene composites *via* a hydrothermal method (Fig. 2f). In this synthesis approach, ethylene glycol (EG), propylene glycol (PG), FeCl_3_·6H_2_O, polyethylene glycol (PEG, used as a dispersant), sodium acetate (NaAc), and MXene were used. The as-prepared composite exhibited a specific surface area of 76.62 m^2^ g^−1^, which was approximately 18.2 times higher than MXene (4.20 m^2^ g^−1^) (Fig. 2g).

**Fig. 2:**
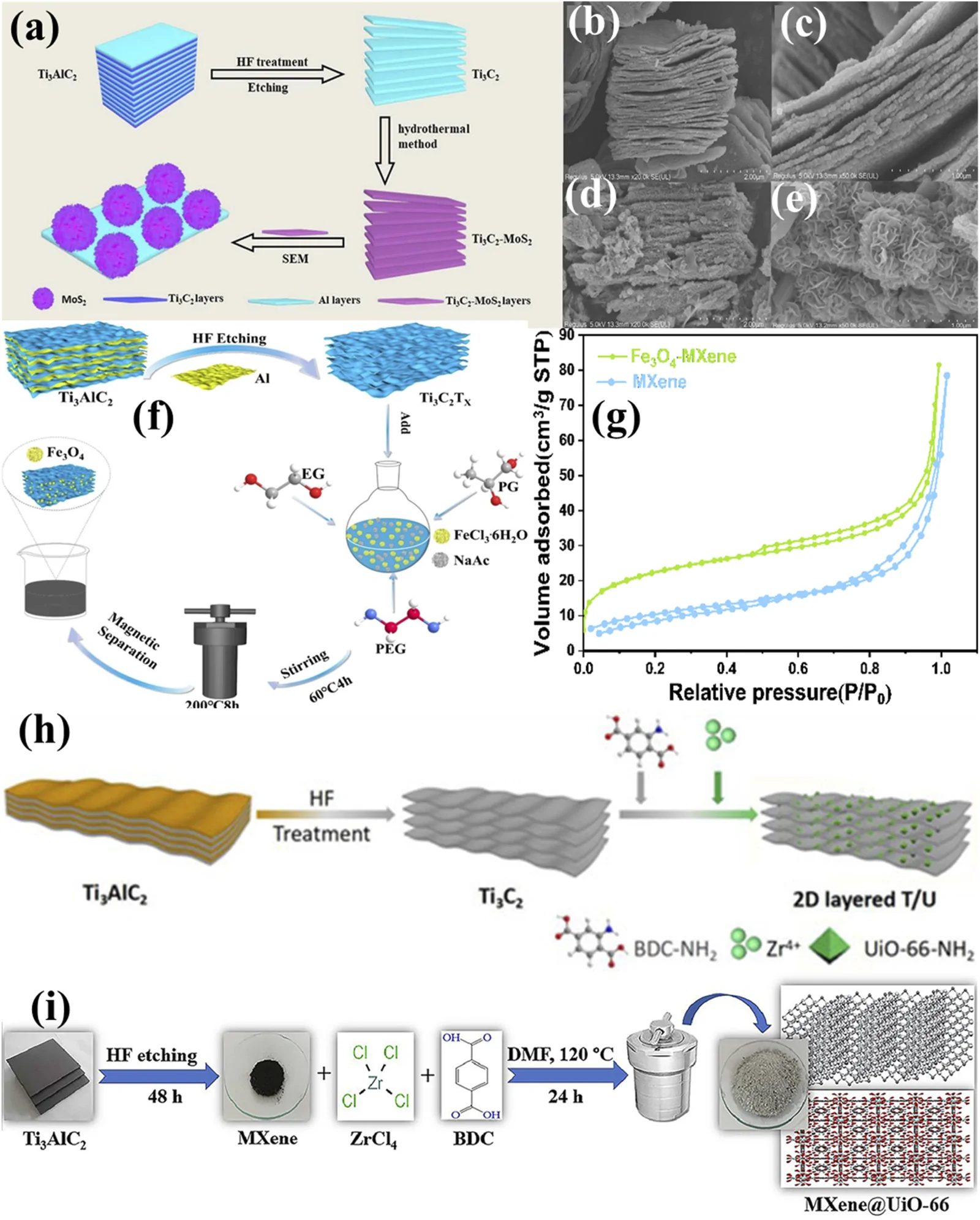
(a) Schematic illustration of the preparation of MXene and MXene–MoS_2_ composite, (b)–(e) SEM micrographs of pristine MXene and MXene–MoS_2_ composite. Figure reproduced with permission from ref. 57, Copyright 2022, Elsevier. (f) Schematic of the synthesis pathway of the Fe_3_O_4_–MXene composite, (g) N_2_ adsorption–desorption isotherms of MXene and Fe_3_O_4_–MXene composite. Figure reproduced with permission from ref. 58, Copyright 2026, Elsevier. (h) Synthesis illustration of MXene and MXene/UiO-66-NH_2_ composites. Figure reproduced from ref. 59, which is open access and permits unrestricted use of materials under the terms of the Creative Commons CC-BY, and (i) preparation of MXene@UiO-66. Figure reproduced with permission from ref. 60, Copyright 2024, Elsevier.

### 2.2 *In situ* method

The spontaneous synthesis of the composite leads to strong interfacial bonding and simplifies the preparation phenomena *via* eliminating complex multi-step routes.^61^*In situ* synthesis is considered one of the most extensively used methods for the preparation of composites.^50^ For instance, He *et al.*^59^ used an *in situ* method for the fabrication of MXene/UiO-66-NH_2_ composites (Fig. 2h). Initially, ZrCl_4_ was dispersed in dimethylformamide (DMF), with acetic acid serving as a modulator during the MOF synthesis, followed by the addition of a specific amount of MXene. The reaction mixture was stirred for 1 h, followed by the addition of DMF solution of pre-dissolved BDC-NH_2_, and the combined solution was further stirred for 30 min. Subsequently, the above mixture was poured into autoclave and maintained at 120 °C for one day to facilitate crystallization. After natural cooling to normal temperature, the yellow precipitate was washed with DMF and anhydrous methanol. The resulting product was vacuum-dried overnight at 80 °C, and the samples were named as 1-T/U, 2-T/U, 3-T/U, and 4-T/U based on MXene contents of 5, 10, 15, and 20 wt%, respectively. Similarly, Far *et al.*^60^ synthesized an MXene@UiO-66 nanocomposite *via* an *in situ* method. The process involved the uniform growth of UiO-66 crystals on MXene nanosheets (Fig. 2i). Initially, ZrCl_4_ and TPA ligands were dissolved in DMF at room temperature with MXene powder, followed by sonication for 15 min. Subsequently, the resulting solution was placed in an autoclave and maintained at 120 °C for 24 h. After natural cooling, the grey precipitates were collected and washed with deionized (DI) water three times. The as-synthesized material was then dried at 80 °C for 6 h.

It is beneficial to distinguish hydrothermal/solvothermal approaches from *in situ* synthesis. Hydrothermal/solvothermal approaches refer the reaction conditions, using water or an organic solvent under high pressure and temperature, respectively, while *in situ* preparation method describes the direct growth of one component in the presence of another component. Thus, *in situ* synthesis can also be performed under hydrothermal/solvothermal conditions.

### 2.3 Self-assembly approach

Nanoscale self-assembly is widely observed in nature, where molecules spontaneously organize into complex structures including cell membranes, biopolymer fibers, and viruses. Recently, researchers have successfully synthesized a variety of nanomaterials in laboratories through a bottom-up approach known as self-assembly. Self-assembly is a process involving the spontaneous arrangement of nanomaterials into organized superstructures. Furthermore, electrostatic self-assembly is one of the most extensively used techniques for the fabrication of nanocomposites.^37^ For instance, Liu *et al.*^62^ prepared Ti-MOF/QDs/ZIS nanocomposite through an electrostatic self-assembly approach (Fig. 3a). Initially, Ti-MOF/ZIS was dispersed in distilled water through ultrasonication, followed by nitrogen bubbling for 60 min to remove dissolved oxygen. Subsequently, an appropriate amount of MXene quantum dots was added to the oxygen-free dispersion. Under neutral pH conditions, MXene QDs possess a negative surface charge, while the Ti-MOF/ZIS surface carries a positive charge, resulting in electrostatic assembly. Thereafter, the mixture was continuously stirred at 35 °C for 12 h under an N_2_ atmosphere. The as-synthesized Ti-MOF/QDs/ZIS nanocomposite was recovered *via* filtration and washed three times with DI water. The remaining solvent was evaporated through vacuum distillation to obtain the final product. In another study, Mishra *et al.*^63^ employed an electrostatic self-assembly approach for the synthesis of 1D/2D MXene–TiO_2_ composite (Fig. 3b). Field emission-scanning electron microscopy (FE-SEM) analysis revealed an accordion-like multilayer structure of MXene, which is associated with a high surface area enlarged interlayer spacing. According to X-ray diffraction (XRD) analysis, the as-prepared composite exhibited good crystallinity and phase purity, indicating the combination of TiO_2_ nanotubes (Fig. 3c). High resolution-transmission electron microscopy (HR-TEM) analysis demonstrated an average particle size of 38.41 nm. Furthermore, X-ray photoelectron spectroscopy (XPS) analysis confirmed the presence of strong interfacial coupling between TiO_2_ nanotubes and MXene sheets.

**Fig. 3:**
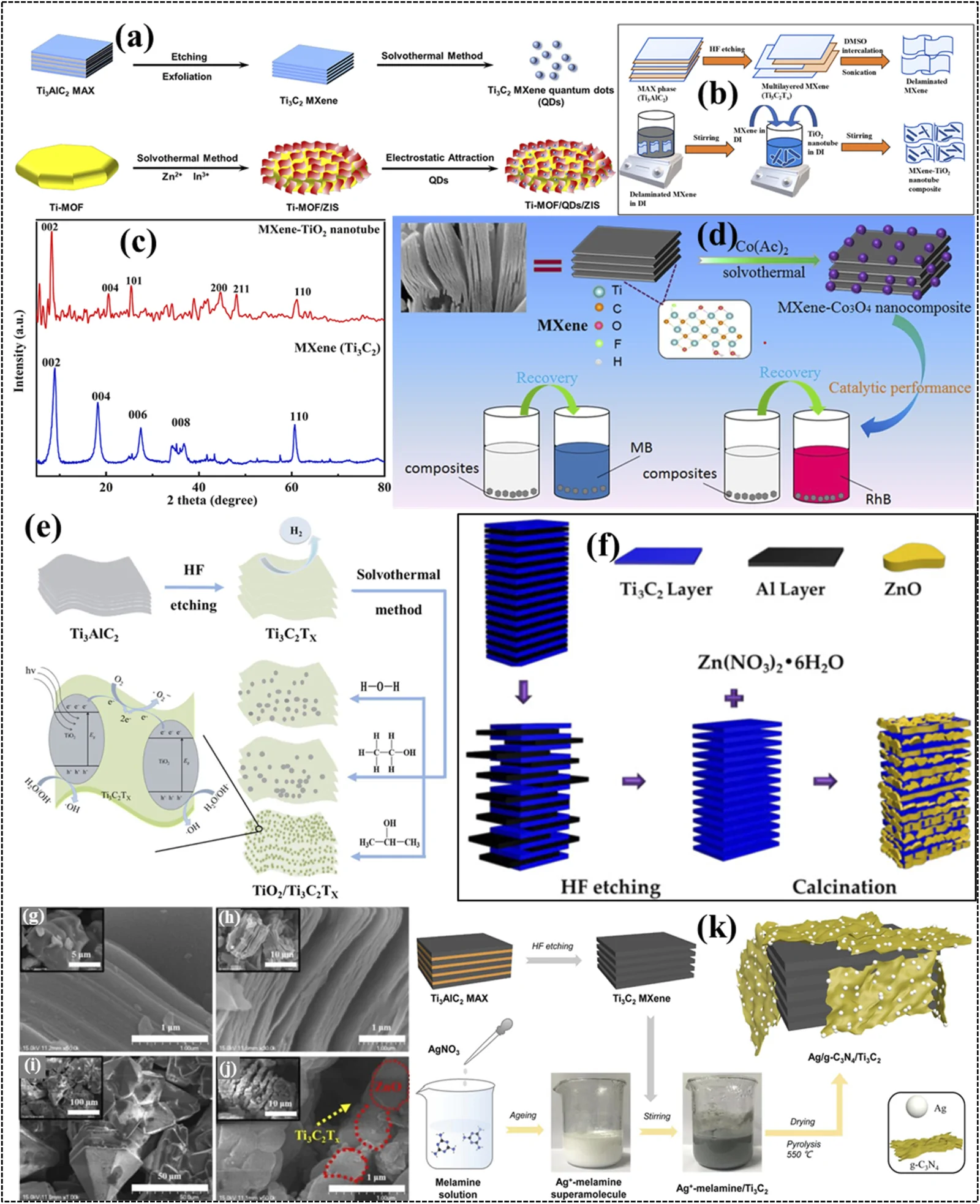
(a) Schematic showing the synthesis process for the Z-scheme NH_2_-MIL-125 (Ti)/MXene quantum dots/ZnIn_2_S_4_ composites (Ti-MOF/QDs/ZIS) synthesis. Figure reproduced with permission from ref. 62, Copyright 2022, Elsevier. (b) Illustration diagram of the preparation of MXene and MXene–TiO_2_ nanotube (TNT), (c) XRD spectra of MXene and MXene–TiO_2_ nanotube. Figure reproduced with permission from ref. 63, Copyright 2024, Elsevier. (d) MXene–Co_3_O_4_ nanocomposites synthesis and application in wastewater treatment. Figure reproduced from ref. 64, which is open access and permits unrestricted use of materials under the terms of the Creative Commons CC-BY (e) synthesis representative process of the TiO_2_/MXene composites. Figure reproduced with permission from ref. 65, Copyright 2014, Elsevier. (f) Schematic illustration of the synthesis process of ZnO/MXene hybrid structures. SEM images of (g) MAX phase, (h) MXene, (i) ZnO, and (j) ZnO/MXene composite,^66^ (k) schematic illustration of Ag/g-C_3_N_4_/MXene composites synthesis process. Figure reproduced from ref. 67, which is open access and permits unrestricted use of materials under the terms of the Creative Commons CC-BY.

### 2.4 Solvothermal method

The solvothermal method is similar to that of hydrothermal synthesis, except that organic solvents (*i.e.*, ethanol, methanol *etc.*) are used instead of water. When glycerol and alcohol are employed as solvents, the processes are referred to as glycothermal and alcohothermal, respectively. The solvothermal is highly efficient and commonly used approach for the fabrication of MXene-based materials, leading to a variety of morphological structures.^37^ For example, Luo *et al.*^64^ a facile solvothermal method was employed to synthesize sheet-like MXene–Co_3_O_4_ nanocomposites (Fig. 3d). In the first step, MXene–Co_3_O_4_ composites were prepared, in which MXene was synthesized from Ti_3_AlC_2_ MAX-phase ceramics through HCl/LiF etching under ultrasonication. In the second step, the prepared materials were employed for the catalytic degradation of methylene blue and rhodamine B. In this study, MXene–Co_3_O_4_ and Co_3_O_4_ were chosen as catalysts for dye degradation.^64^ For instance, Tian *et al.*^68^ synthesized a MXene/TiO_2_/UiO-66-NH_2_*via* a solvothermal process. The synthesis process involved stirring the ZrCl_4_ solution for 1 h, followed by the addition of 2 wt% TCA and subsequent addition of BDC-NH_2_ after stirring for 30 min. The above mixture has been poured into autoclave and heated for one day at 120 °C. After cooling at room temperature, the precipitates were washed with DMF to eliminate residual organic ligands, followed by solvent exchange using anhydrous methanol. The resulting yellow MXene/TiO_2_/UiO-66-NH_2_ product was separated by centrifugation, followed by vacuum-drying at 80 °C for 12 h. In another study, Chen *et al.*^65^ prepared a TiO_2_/MXene composite *via* a solvothermal method using different chemical reagents (Fig. 3e). Under light irradiation, the photogenerated excitations were effectively separated across the MXene nanosheets, thereby enhancing the photocatalytic performance. Overall, solvothermal synthesis provides a versatile route for integrating MXene with metal oxides and MOF-derived components, particularly when controlled nucleation and intimate interfacial contact are required. However, the use of organic solvents, relatively long reaction times, and autoclave-based processing can increase the environmental and operational burden compared with aqueous routes. Therefore, the selection of solvothermal conditions should consider not only composite formation but also solvent compatibility, interfacial quality, and scalability.

### 2.5 Calcination

In the calcination method, materials are subjected to high temperatures under controlled conditions. It has been extensively used for the fabrication of nanomaterials.^37^ For instance, Ta *et al.*^66^ employed a calcination approach for the fabrication of a ZnO/MXene composite (Fig. 3f). The bandgap energy and specific surface area of ZnO/MXene composite was found to be 1.6 eV and 2.48 m^2^ g^−1^, respectively. Morphological analysis revealed the formation of rice crust-like ZnO structures anchored on MXene sheets (Fig. 3g–j). Similarly, Huang *et al.*^67^ used a one-step calcination method for the synthesis of Ag/g-C_3_N_4_/MXene nanocomposites (Fig. 3k). Owing to strong interfacial heterojunction contact, the introduction of Ag nanoparticles facilitated localized surface plasmon resonance and enhanced visible-light activity. The resulting materials displayed wrinkled and bent layered morphologies. Moreover, the layered MXene acted as an electron acceptor and co-catalysts, facilitating efficient charge separation and O_2_ adsorption, which inhibited electron–hole recombination and improved photocatalytic performance.

## 3. MXenes families and their structural features

The compositional diversity of MXenes across the periodic table is revealed in Fig. 1c.^43^ MXenes obtained from MAX phases generally exhibit structural formulas such as M_2_C, M_3_C_2_, and M_4_C_3_. Double transition metal MXenes are majorly categorized into two classes: ordered double transition metal MXenes and solid-solution MXenes. Ordered double transition metal MXenes generally follow the formula 
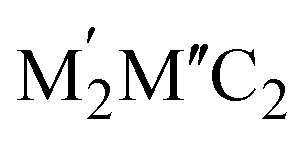
 or 
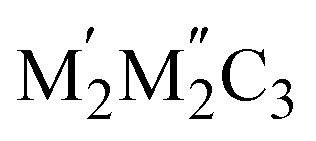
, where M′ and M″ represent two different transition metals. In contrast, solid-solution MXenes typically follow the formulas 
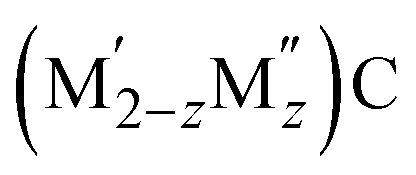
, 
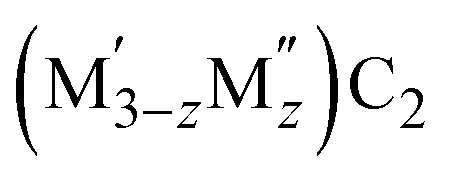
, 
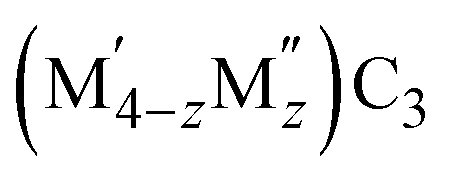
, or 
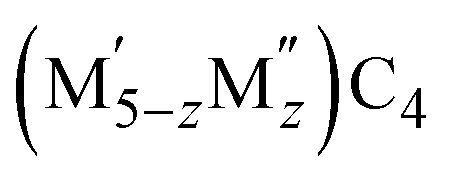
. Their properties can be continuously tuned due to the random arrangement of transition metal atoms within the crystal lattice.^69^ Moreover, besides random solid solutions at the M-site, MAX phases containing chemically ordered transition metals have attracted considerable scientific attention in recent years.^70^ The first experimentally recognized chemically ordered MAX alloy was (Cr_2/3_Ti_1/3_)_3_AlC_2_.^71^ This structure can be simplified as M_3_AX_2_, consisting of alternating atomic layers containing a single transition metal and mixed layers with two transition metals in a ratio of 2 : 1, resulting in out-of-plane chemical ordering. Subsequently, another out-of-plane ordered structure with an M_4_AX_3_ configuration, namely Mo_2_Ti_2_AlC_3_, was reported, in which two Mo layers sandwich two Ti layers, and the corresponding MXene, Mo_2_Ti_2_C_3_T_*x*_, was obtained by selective etching of Al.^72^ In addition, MXene sheets containing controlled metal divacancies (Mo_1.33_C) can be synthesized from specially designed three-dimensional laminated precursors with in-plane chemical ordering, (Mo_2/3_Sc_1/3_)_2_AlC, by selectively etching both Al and Sc atoms.^73^ This precursor displays in-plane chemical ordering, and the selective etching of Al and Sc atoms produces a new class of structures referred to as I-MAX phases.^73^ Similar to their parent MAX phases, MXenes consist of closely packed hexagonal crystal structures, as demonstrated in Fig. 4a.^74,75^ The transition metal atomic layers are arranged in an XYXYXY stacking sequence, whereas M_3_X and M_4_X structures exhibit an XYZXYZXYZ stacking order (Fig. 4b).^75^ In general, double-transition-metal MXenes contain two different transition metals and are mainly classified into ordered phases and solid-solution phases. In ordered MXenes, one transition metal is sandwiched between layers of another transition metal, as predicted and observed in compounds such as Cr_2_TaC_2_, Mo_2_V_2_C_3_ and Mo_2_NbC_2_.^76^ In contrast, solid-solution MXenes contain two transition metals that are randomly distributed within the M layers, with examples including (Ti, Nb)_2_C, (Nb, Zr)_4_C_3_ and Ti_3_CN.^77,78^ Advanced theoretical simulations and computational studies have extrapolated that the M atoms in MXenes occupy hexagonal close-packed lattice positions, while the X atoms are located at octahedral interstitial sites. The presence of surface functional groups on MXenes has been experimentally confirmed through techniques such as X-ray photoelectron spectroscopy (XPS) and energy dispersive spectroscopy (EDS). Among the M_2_C-type MXenes, M_1.33_C is unique because it contains about 33% vacancies within the transition metals layers. In addition, density functional theory (DFT) calculations suggest that ordered MXene phases possess greater structural stability with respect to disordered solid solution structures. The thickness and number of layers in MXenes have been examined using high-angle annular dark-field scanning transmission electron microscopy (HAADF-STEM) which provides detailed visualization of their layered morphology.^79^

**Fig. 4:**
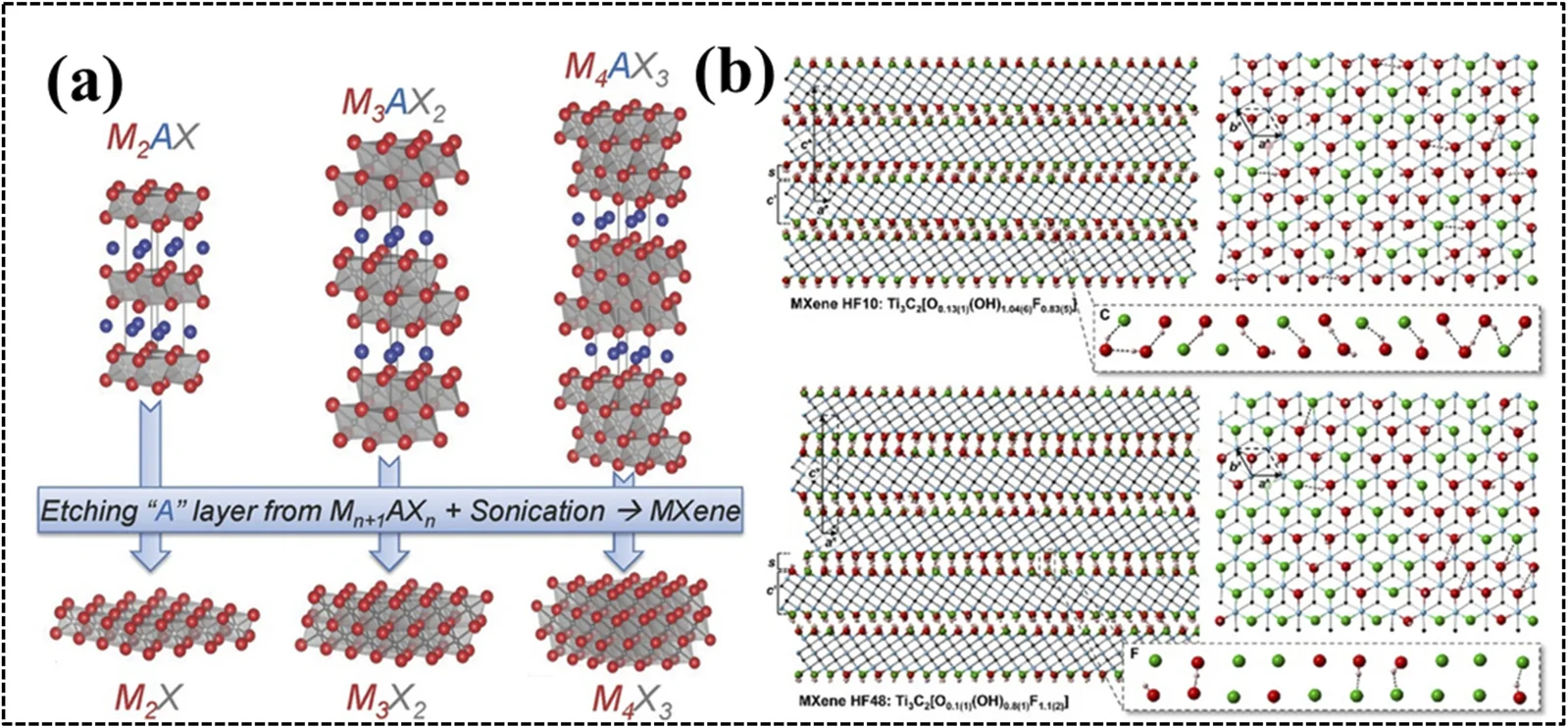
(a) Schematic representation of crystal structures of MAX phases and their corresponding MXene sheets, (b) surface functional group composition of MXene as a function of HF concentration. HF10 and HF48 represent the concentrations of aqueous HF solutions (10% and 48% HF, respectively) used for etching the aluminum layer. Figures reproduced with permission from ref. 75 Copyright 2022, Elsevier.

MXenes consist of hexagonally close-packed structures that closely resemble those of their parent MAX phases, from which the A layer is removed by selective etching.^80^ Theoretical investigations have revealed that these surface terminations (–O, –OH, –F) strongly influence the electronic structure and electrochemical behavior of MXenes.^36,81^ Furthermore, the presence of such functional groups improves hydrophilicity and metallic conductivity, making MXenes highly promising materials for energy storage applications. The type and distribution of surface terminations mainly depend on the synthesis route employed during MXene preparation.^82^ Moreover, these terminations can be further improved through post-treatment processes. Besides surface chemistry, the interlayer spacing of MXenes can also be tuned. For example, Luo and co-workers achieved a 177% expansion in the interlayer spacing of MXene by using cationic surfactants as intercalating agents.^83^

The electrical conductivity of MXenes is strongly affected by both the type of surface terminations and the transition metal composition.^84^ For instance, oxygen-containing terminations (–O) adsorbed on the surface of MXene can reduce the Fermi energy, resulting in the transformation of metallic MXene into semiconducting Ti_3_C_2_O_2_. In contrast, fluorine terminations (–F) also lower the Fermi energy of MXene, but the resulting MXene-based structure largely retains its metallic nature. The choice of transition metal elements also plays a crucial role in determining the conductivity of MXenes. For example, Mo_2_Ti_2_C_3_T_*x*_ shows higher electrical conductivity than Mo_2_TiC_2_T_*x*_; however, both materials possess significantly lower conductivity compared with MXene. These observations clearly demonstrate the substantial influence of transition metal composition on the electronic transport properties of MXenes.^82^

## 4. Properties of MXene and MXene-based materials

MXene and MXene-derived materials with their exceptional and unique characteristics have drawn attention in material science. These materials bear remarkable properties such as photocatalytic efficiency, structural stability, electrical conductivity, biocompatibility, and optical performance, making them suitable for advanced technological applications. From a critical perspective, the properties of MXene-based materials are highly dependent on their composition, surface chemistry, morphology, and interactions with the coupled components. Therefore, these properties should be considered collectively when assessing their photocatalytic performance, as improvements in one property may not necessarily result in enhanced overall photocatalytic activity. This part of the study discusses the major properties of MXene and MXene-based materials in detail (Fig. 5).

**Fig. 5:**
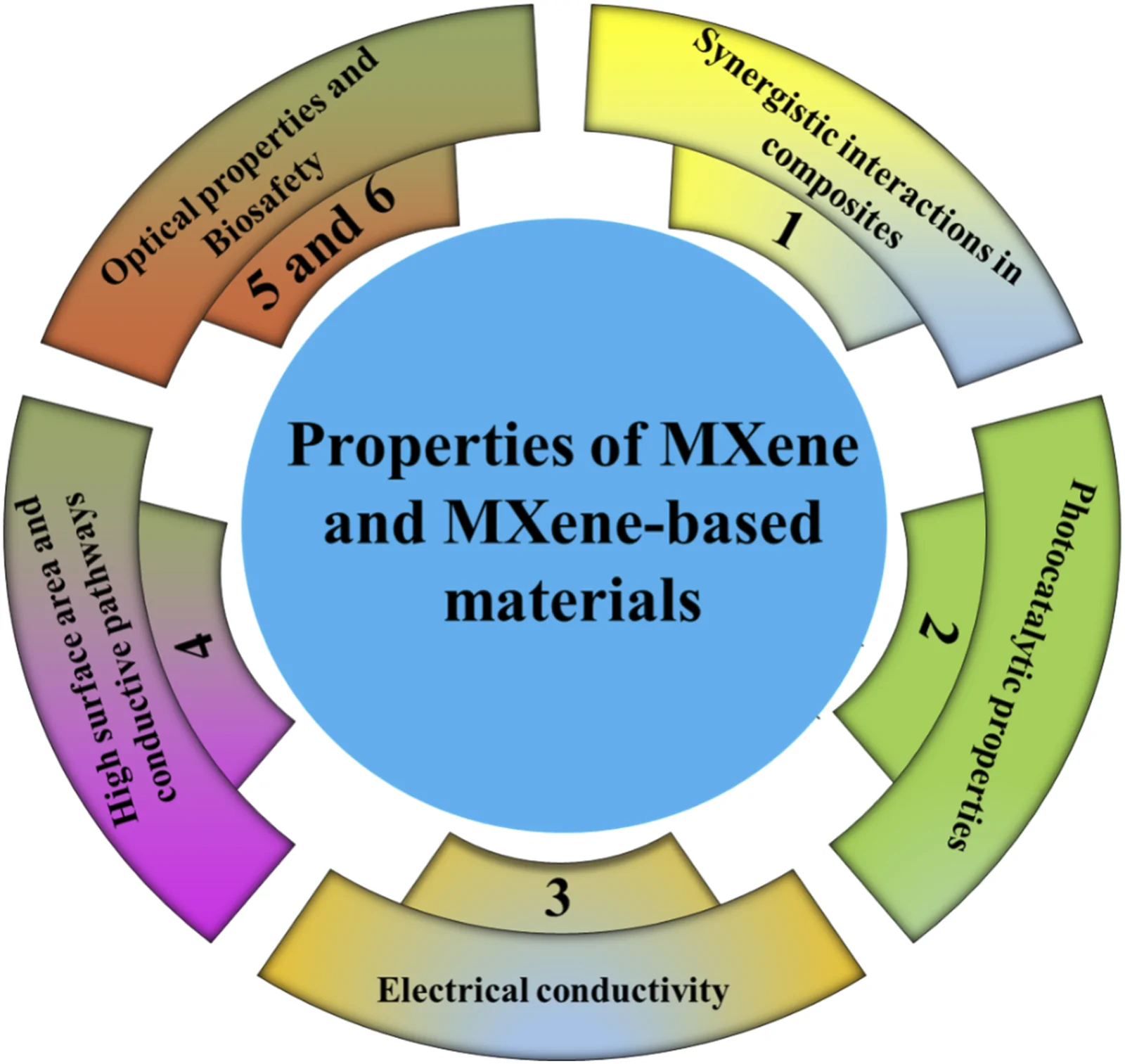
Different properties of MXene and MXene-based materials.

### 4.1 Synergistic interactions in composites

The adaptable nature of MXenes allows their integration with distinct classes of materials, including metal sulfides, metal oxides, conductive polymers, and carbon nanostructures, resulting in hybrid composites with increased functionality. The enhanced performance of these composites originates from combined interaction between the constituent materials. For instance, MXenes offer excellent conductivity and mechanical stability, whereas other constituents may contribute enhanced electrochemical activity or high charge-storage capability. Within these hybrid systems, the MXenes generally function as structural support as well as conductive framework. MXenes facilitate rapid electron transfer, reduce structural degradation because of volume expansion, and promote uniform distribution of active species. The magnitude of this effect depends strongly on MXene composition and surface termination; for example, reported MXene work functions range from approximately 1.8 to 8.0 eV, highlighting the substantial tunability of their electronic properties.^85,86^ Moreover, strong interfacial interactions of MXenes with accompanying composite materials enhance ion diffusion, speeding up redox processes, and improves overall electrochemical stability. As a result, these synergistic effects support many lithium-storage mechanisms, including conversion reactions, ion intercalations, capacitive behavior, and surface redox activity. Hence, MXene-based nanomaterials often show superior specific capacity, longer cycling durability, and faster rate capability compared with individual component material alone.^39,87,88^

### 4.2 Photocatalytic properties

MXene-based materials have remarkable physiochemical properties which attracted considerable attention in the photocatalysis.^89^ The abundance of surface functional group and interlayer spacing making these materials highly efficient photocatalysts in different photocatalytic processes.^90^ Furthermore, mechanical stability and large surface area are the factors which improves the photocatalytic ability of the MXene-derived materials.^34^ The superior photocatalytic behavior of MXene-derived materials can be illustrated by several important factors. First, the MXene surfaces contain many –O and –OH functional groups which promote maximum interfacial interaction with photocatalytic materials. Second, the high electrical conductivity and tunable electronic structure of MXenes promote charge-carrier transport in photocatalytic heterostructures. This effect is particularly important for Ti_3_C_2_T_*x*_-based composites, where interfacial interactions with semiconductor components can suppress electron–hole recombination and promote electron transport, thereby contributing to enhanced photocatalytic activity.^91^ Third, the exposed terminal metal atoms on the surfaces of MXenes facilitates stronger redox activity as compared to conventional carbon-based materials. Moreover, their excellent hydrophilic nature improves interaction with water molecules, which is useful for aqueous photocatalytic reactions. MXenes also act as an efficient photocatalysts which supports mass-transfer media, and offers superior semiconductor anchoring capability and dispersion while generating nanoscale transport pathways for reactants.^92,93^ Among different MXene constituents, MXene has drawn attention in the field of photocatalysis because of its relatively low Fermi energy level and large amount of surface termination groups.^94^ In addition, the interfacial interaction occurred between inorganic semiconductors and MXene creates a favorable band alignment that reduces charge-carrier recombination significantly enhances photocatalytic efficiency.^95^ For example, MXene–TiO_2_ have showed improved photocatalytic performance in degradation of dye because of the synergistic interaction between both constituent.^96,97^ In this hybrid system, MXene minimizes charge recombination and promotes efficient electron transport, whereas TiO_2_ offers highly active photo-responsive sites in the presence of UV-light irradiation.^98^ The high interfacial interaction between MXene and TiO_2_ further facilitates the generation and transfer of charge carriers, resulting in decreased charge recombination and enhanced photocatalytic activity.^99,100^

### 4.3 Electrical conductivity

The photocatalytic performance of MXene and MXene-derived materials is closely linked with their electronic properties because photocatalytic reactions strongly depend on the transfer, separation, and generation of light-induced charge carriers. Therefore, understanding and controlling the electronic properties of MXenes is necessary for the development of highly potent MXene-based photocatalysts.^101^ The electronic behavior of MXenes can be significantly affected by both their transition metal constituents and surface terminal groups. Changing these surface terminations can change MXenes from metallic conductors into semiconductors or even topological insulators.^102^ In the previous study, Liu *et al.*^103^ demonstrated the work functions of metallic MXenes and reported a broad range from 1.8 to 8 eV. This broad variation illustrates the strong dependence of MXene electronic properties on surface chemistry. For a given MXene, the incorporation of –OH surface groups generally minimize the work function, while –O terminations tend to increase it, relative to the pristine surface. Subsequently, –F terminations produce intermediate or material-dependent effects. Notably, all OH-terminated MXenes showed exceptionally low work function below 2.8 eV, which is even lower than scandium (contain the smallest work function among elemental metal). In contrast, certain O-terminated MXenes showed work functions higher than platinum (known for having highest work function among elemental metal). Meanwhile, the work function of F-terminated MXenes generally lies between those of the –OH and –O functionalized counterparts. These changes in work functions are associated with the variations in surface dipole moments induced by functionalization. Specifically, –OH group produce negative surface dipole moments that decreases the work function, whereas –O groups generate positive dipole moments that increases work function. Overall, the art to tune the electrical and electronic properties of MXene through surface functionalization offers an effective approach for improving photocatalytic performance and vesting their potential applications in solar energy harvesting and environmental remediation.^37^

### 4.4 High surface area and conductive pathways

MXene shows 2D layered structure characterized by large specific surface area and excellent intrinsic electrical conductivity.^39,104^ This unique architecture offers the formation of continuous electron transport pathways within the electrode matrix, hence decreasing internal resistance and improving charge-carrier mobility. When coupled with composite material, MXenes generate interconnected conductive networks that promote efficient electron transfer between electroactive particle and the current collector. Moreover, the extensive surface area of MXenes supports the uniform distribution of active materials, which enhances electron transport uniformity and improves the electrochemical utilization of the active components.^39,105^ Their high surface-to-volume ratio also enables better electrolyte diffusion and more efficient ion exchange at the electrode–electrolyte interface. Therefore, MXene-derived composite shows reduced polarization, superior photocatalytic performance, and enhanced rate capability.^39^

### 4.5 Optical properties

MXene-derived materials reveal significant optical properties, making them potent candidate for applications in photovoltaic systems, optoelectronic devices, and photocatalysis.^41,106^ Thin films of MXene illustrate strong light absorption in the UV-region, particularly within the range of 300–500 nm.^107^ Moreover, these films display a wide and intense adsorption around 700–800 nm, which can be adjusted by controlling film thickness and through ion intercalation processes.^108^ Such tunable optical behavior enables the fabrication of light-responsive green films suitable for photothermal applications. The optical and electromagnetic performance of MXene-derived composites is also strongly influenced by their microstructure. For instance, in NiCo-layered double hydroxide (NiCo-LDH)/MXene hybrids, variations in MXene content significantly change the functionality and morphology of the composite. At a MXene loading of 50 wt%, the Nico-LDH structure extensively covers the MXene sheets. When the MXene content increases to 66 wt%, the NiCo-LDH structures become attached to the MXene surfaces, further increase in the MXene concentration to 75% leads to the Nico-LDH flower-like structures being distributed between MXene layers, producing an interconnected network that improves electromagnetic wave absorption. However, at 80 wt% MXene content, the NiCo-LDH morphology becomes less differentiable, suggesting the attainment of a structural saturation point within the material.^109^ Overall, the optical properties of MXene-derived materials can be effectively personalized through structural and compositional engineering, thereby expanding their potential applications in light-harvesting systems, advanced photonic devices, and photothermal technologies.

### 4.6 Biosafety

The biocompatibility and safety assessment of MXene-based materials are important for their successful applications in the biomedical field. Assessing the factors such as cellular uptake mechanism, immune responses, interaction with DNA, and the potential to induce oxidative stress is crucial for understanding the biological impacts of these materials. Numerous studies have suggested that MXenes may induce cellular damage through oxidative stress generation, interference with intracellular signaling pathways, and membrane disruption. Certain investigators have confirmed the adverse effects of MXenes on zebrafish embryos, denoting their usefulness as biological models for nanotoxicity evaluation.^37^ Irrespective of the above findings, MXenes have illustrated promising biocompatibility in mammalian systems. For instance, Pan *et al.*^110^ observed the influence of three-dimensional MXene-bioglass scaffolds on bone development in rats. After 6 months of implantation, no significant hematological or histological abnormalities were observed compared with the control group, showing minimal toxic effects. In another investigation, a high-throughput toxicity assessment was carried to evaluate the biosafety of Nb-based MXenes using zebrafish embryos as the experimental model.^111^ Acute toxicity study demonstrated complete mortality at a concentration of 300 mg L^−1^ for all tested nanosheets, consisting multi layered ML-Nb_2_CT_*x*_ and ML-Nb_4_C_3_T_*x*_ as well as delaminated DL-Nb_2_CT_*x*_ and DL-Nb_4_C_3_T_*x*_, the calculated LC_50_ values after 96 hours post-fertilization were obtained to be 20 mg L^−1^ for ML-Nb_2_CT_*x*_, 215 mg L^−1^ for ML-Nb_4_C_3_T_*x*_, 225 mg L^−1^ of DL-Nb_2_CT_*x*_, and 128 mg L^−1^ of DL-Nb_4_C_4_T_*x*_, in addition lactate dehydrogenase (LDH) release assays illustrated greater membrane damage in DL-Nb_4_C_3_T_*x*_ compared with DL-Nb_2_CT_*x*_. Nevertheless, both delaminated Nb-derived MXene nanosheets demonstrated no significant adverse effects on hepatic or cardiac functions at the no observed effect concentration (NOEC) showing their potential suitability for biomedical applications under controlled exposure conditions.^112^ From a photocatalytic perspective, biosafety is important because MXene-based photocatalysts may remain in treated water or release components during and after the degradation process. Therefore, evaluating their potential toxicity is essential to ensure that the treatment process does not introduce secondary environmental risks. A photocatalyst should ideally combine high pollutant degradation efficiency with good environmental compatibility and stability.

## 5. Factors affecting the photocatalytic degradation of organic pollutants using MXene-based materials

The efficiency of photocatalytic removal of organic contaminants through MXene-based catalysts is strongly influenced *via* several operational parameters, including photocatalyst amount, initial pollutant concentration, pH of the solution, temperature, and the amount of oxidants, including hydrogen peroxide (H_2_O_2_), persulfate (PS), and peroxymonosulfate (PMS) (Fig. 6a).^42^ Therefore, careful optimization of these parameters is crucial to obtaining highest degradation efficiency and enable practical environmental applications. Tables 2 and 3 shows the photocatalytic activity of different MXene-based materials toward the degradation of various organic contaminants. These studies demonstrate that these catalytic systems show outstanding performance in removing a wide range of pollutants, such as carbamazepine, sulfamethoxazole, atrazine, rhodamine b, naproxen, and ranitidine.

**Fig. 6:**
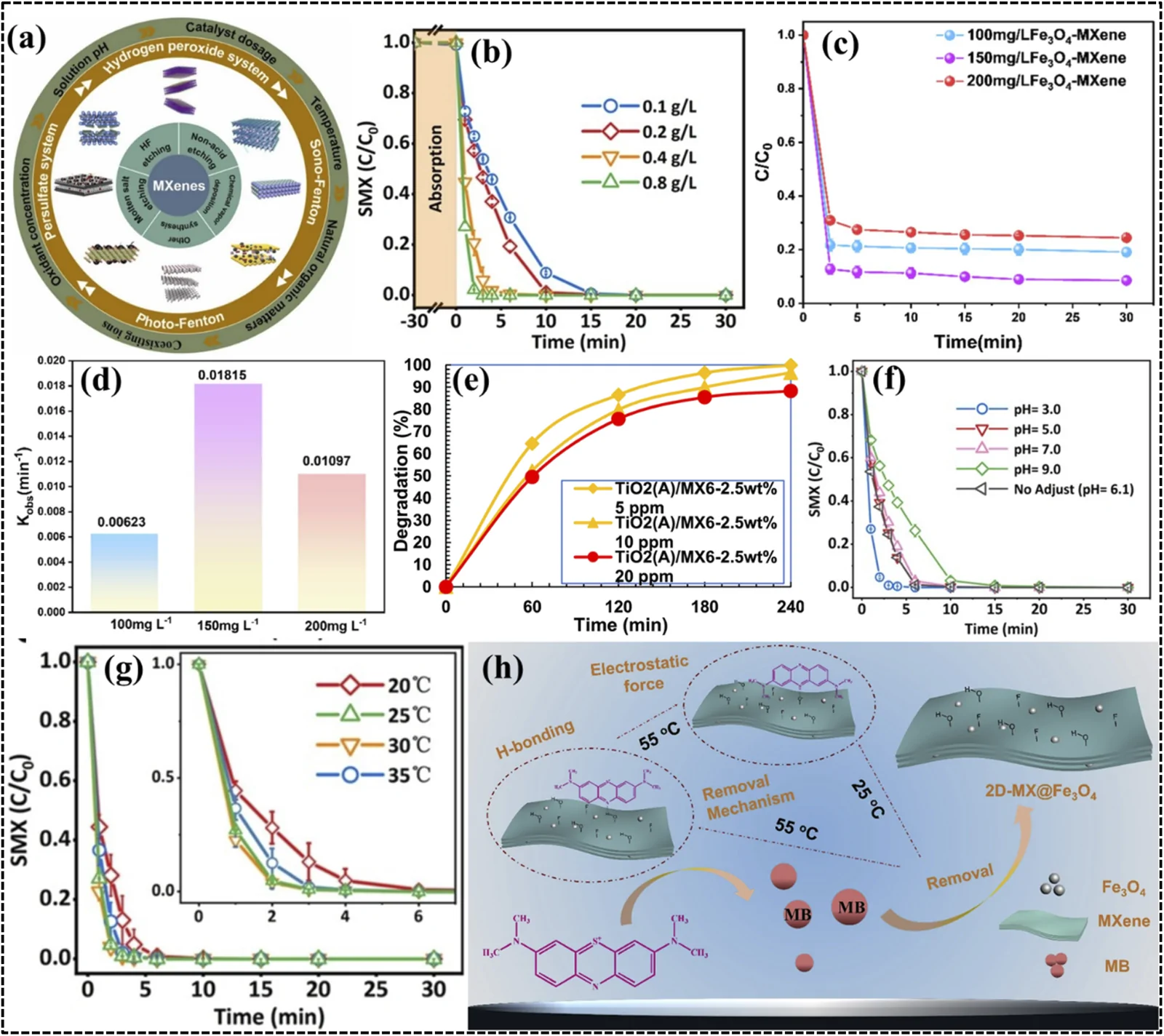
(a) Factors affecting the removal of organic contaminants in the presence of MXene-based materials. Figure reproduced with permission from ref. 42, Copyright 2022, Elsevier. (b) Effect of various MXene/Fe_3_O_4_ concentrations on SMX elimination. Figure reproduced with permission from ref. 113, Copyright 2024, Elsevier, (c) effect of photocatalyst dosage on tetracycline degradation and (d) its *k*_obs_ values. Figure reproduced with permission from ref. 58, Copyright 2026, Elsevier. (e) Effect of phenol red (organic pollutants) concentration on its removal in the presence of TiO_2_/MXene-2.5 wt%. Figure reproduced from ref. 114, which is open access and permits unrestricted use of materials under the terms of the Creative Commons CC-BY (f) impact of pH on degradation of SMX by MXene/Fe_3_O_4_,^113^ effect of various temperature on the degradation of (g) SMX. Figure reproduced with permission from ref. 113, Copyright 2024, Elsevier, and (h) methylene blue (MB). Figure reproduced with permission from ref. 115, Copyright 2019, Elsevier.

**Table 2:** Summary of the key findings related to the photocatalytic degradation of organic pollutants *via* MXene-based materials in the presence of oxidant

Photocatalyst	Dosage	Synthesis approach	Contaminant (concentration)	Oxidant (concentration)	Surface area (m^2^ g^−1^)	Bandgap (eV)	Light source	Reaction conditions		Results		Reference
								pH	Time (min)	% Degradation	*k*_obs_ (min^−1^)	
CuS@MXene	0.5 g L^−1^	Hydrothermal treatment	O-II (60 mg L^−1^)	PS (0.2 g)	19.34	—	LED incandescent lamp (120 W)	—	60	100	0.02633	116
MXene/CuFe_2_O_4_	500 mg L^−1^	*In situ* chemical co-precipitation followed by calcination	OFL (10 mg L^−1^)	PMS (100 mg L^−1^)	—	—	Xe lamp (300 W)	6	90	88.0	0.0242	117
MXene/CuFe_2_O_4_	500 mg L^−1^	*In situ* chemical co-precipitation followed by calcination	OFL (10 mg L^−1^)	PMS (100 mg L^−1^)	—	—	Absence of light	6.0	90	73.0	—	117
MXene/g-C_3_N_4_ (1.5 MCN)	0.1 g L^−1^	Thermal exfoliation	SMX (5 mg L^−1^)	PS	108.5	2.58	300 W xenon lamp (400 nm optical filter)	—	20	98	0.195	118
MXene/g-C_3_N_4_ (1.5 MCN)	0.1 g L^−1^	Thermal exfoliation	SDZ^a^ (5 mg L^−1^)	PS	108.5	2.58	300 W xenon lamp (400 nm optical filter)	—	20	97.7	0.195	118
ZnFe_2_O_4_/MXene	0.05 g	Hydrothermal approach	TC (30 mg L^−1^)	PS (0.75 g L^−1^)	—	—	120 W LED incandescent lamp	—	60	87	0.0328	119
MXene/Fe^3+^	60 mg L^−1^ of MXene and 3.35 mg L^−1^ of Fe^3+^	—	RR218 (0.05 g L^−1^)	Sodium PS (1–4 g L^−1^)	—	—	—	3.0	30	98.5	0.13	120
MXene-derived TiO_2_–Ce_2_Sn_2_O_7_	50 mg/100 mL	Thermal oxidation followed by coupling	PFOA (4 mg L^−1^)	H_2_O_2_	232	2.28	300 W xenon lamp with (420 nm filter)	4.0	80	98	0.091	121
N–TiO_2_/Fe_3_O_4_@MXene	0.10 g L^−1^	Hydrothermal method	Phenol (40 mg L^−1^)	H_2_O_2_ (100 mM)	—	—	Xe lamp (300 W)	3.0	30	98	—	122
MXene-supported BiVO_4_/InVO_4_	0.267 g L^−1^	Hydrothermal method	OFX (26.53 mg L^−1^)	H_2_O_2_	40–50	—	300 W Xe lamp	6.63	65.24	99	0.08	123

a SDZ represents sulfadiazine.

**Table 3:** Summary of the key findings related to the photocatalytic removal of organic pollutants *via* MXene-based materials

Photocatalyst	Dosage	Synthesis approach	Contaminant (concentration)	Surface area (m^2^ g^−1^)	Bandgap (eV)	Light source	Reaction conditions		Results		Reference
							pH	Time (min)	% Degradation	*k*_obs_ (min^−1^)	
Pd/MXOF	0.02 g	Hydrothermal followed by green approach	OFL (20 mg L^−1^)	65.87	1.79	LED lamp (175 W m^−2^)	5.0	30	>99	—	124
Sn–Bi-MOF/MXene	40 mg	Solvothermal method	TC (20 mg L^−1^)	145.87	—	Xenon lamp (500 W)	11	90	96.2	—	125
ZnFe_2_O_4_/Cr_2_O_3_/MXene	15 mg L^−1^	Hydrothermal method followed by calcination	TC (50 mg L^−1^)	165	2.65	Xenon lamp (300 W with a 420 nm UV-cutoff filter)	5.0	60	94.2	0.0983	126
Nd/Dy-MOF@MXene	20 mg	Solvothermal	MO (20 mg L^−1^)	55.21	2.78	Sunlight	3.0	30	93.55	0.0989	127
Ti_2_C@GMA/PEDOT:PSS	10 mg L^−1^	Self-assembly with modification	RhB (10 mg L^−1^)	—	—	Visible light	7.0	60	94.9	0.00317	128
Ti_2_C@GMA/PEDOT:PSS	10 mg L^−1^	Self-assembly with modification	CR	—	—	Visible light	—	60	98.6	0.01044	128
MXene decorated ZnO-tetrapod (MZT)	50 mg/50 mL	Blending and sonication	MB (10 mg L^−1^)	5.825	2.75	Sunlight	7.0	120	50.83	0.000041	129
MXene decorated ZnO-tetrapod (MZT)	50 mg/50 mL	Blending and sonication	RhB (10 mg L^−1^)	5.825	2.75	Sunlight	7.0	120	77.6	0.00534	129
BiOIO_3_/MXene	0.5 g L^−1^	Hydrothermal method	TC-HCl (10 mg L^−1^)	28.48	—	Xe lamp (300 W)	7.0	120	93.4	—	130
MXene/In_2_S_3_/CeO_2_ (TM_0.025_IC-15%)	50 mg L^−1^	Reflux method	DCF (10 mg L^−1^)	220.173	1.64	Visible light	—	180	99.7	0.03663	131
MXene/TCPP	—	Self-precipitation method	RhB (10 mg L^−1^)	—	—	Xenon lamp (300 W)	—	50	97.5	0.033	132
La-MOF@MXene (LM-0.1)	10 mg	Solvothermal	TC-HCl (10 mg L^−1^)	40.9	—	300 W xenon lamp	6.0	120	92.4	0.00773	133
MX@MCN	0.25 g L^−1^	—	ENR (10 mg L^−1^)	825	2.4	LED lamps (465 ± 40 nm)	7.0	60	99	0.069	134
UNiMOF/MXene	50 mg	Electrostatic self-assembly process	MB (20 mg L^−1^)	27.00	—	Xenon lamp (300 W)	—	120	99.49	0.0413	135
MXene/MOF aerogel MPA-m/U66N-M	0.1 g	Microwave assisted hydrothermal approach	Acetone (C_3_H_6_O)	1205	2.40	Xenon lamp with a vis-light reflecting filter (350–780 nm)	—	60	95.0	0.052	136

### 5.1 Effect of photocatalyst dosage

The number of MXene-based catalysts plays a crucial role in achieving effective removal of organic contaminants. Many studies have showed that the addition of an appropriate amount of catalyst significantly increases photocatalytic performance.^42^ Several studies have evaluated the effect of MXene-based photocatalyst dosage on the degradation of targeted organic contaminants. For example, Huang *et al.*^113^ studied the effect of catalyst amount on sulfamethoxazole (SMX) removal through Fe_3_O_4_/MXene composite. In this study, different amounts of catalyst, such as 0.1, 0.2, 0.4, and 0.8 g L^−1^, were examined to determine the most effective dosage. The results revealed that more than 99.9% removal of targeted pollutant (SMX) was obtained in just 4 min using 0.8 g L^−1^ Fe_3_O_4_@MXene. Similarly, complete degradation was achieved in just 10 and 20 min when photocatalyst dosages were 0.2 and 0.1 g L^−1^, respectively. These investigations clearly show that catalyst dosage strongly affects the removal of SMX. Although, the highest removal rate was observed at 0.8 g L^−1^ weight of the Fe_3_O_4_@MXene. Consequently, 0.2 g L^−1^ was considered the optimum dosage due to providing high removal efficiency of targeted pollutants, with lower material consumption, making the process more economical and environmentally favorable. As illustrated in Fig. 6b, the degradation efficiency of SMX increase noticeably with increasing catalyst dosages. In another study, Gao *et al.*^58^ studied the influence of Fe_3_O_4_–MXene dosage on tetracycline (TC) degradation (Fig. 6c and d). When the catalyst (*i.e.*, Fe_3_O_4_–MXene) amount increase from 100 to 150 mg L^−1^, a substantial enhancement in the removal of TC was obtained. While, the reaction followed pseudo-first-order kinetics, with rate constant value increased from 0.00623 to 0.01815 min^−1^, showing accelerated degradation kinetics. This improvement was mainly attributed to the increased number of available active sites provided by the higher photocatalyst loading, which enhanced both degradation and adsorption of TC. However, further increase in catalyst amount to 200 mg L^−1^ caused a decline in removal efficiency and reaction rate. Furthermore, Akbari *et al.*^137^ reported that the degradation efficiency increased from 68.7 to 97.98% when the Fe_2_O_3_–SiO_2_/MXene dosage raised from initial concentration to 0.7 g L^−1^. However, a negligible enhancement in removal efficiency was detected when the catalyst amount exceeds 0.75 g L^−1^ (*i.e.*, optimal dosage). This lower increase may be due to the decrease of active sites and lower surface area for the adsorption of photon resulting from the agglomeration nanomaterials and the decrease in the penetration rate of light. Generally, increasing the dosage of MXene-based photocatalysts improves pollutant degradation due to the large number of active sites becoming available for adsorption and photocatalytic reactions. However, the amount of catalyst exceeds the optimum level, the % removal of targeted organic compound may decline because of particle aggregation, lowered light penetration, and blockage of active sites. Therefore, evaluating the optimum catalyst amount is require for maximizing removal effectiveness while maintaining economic and environmental sustainability.^42^

### 5.2 Effect of pollutant concentration

The initial concentration of pollutants significantly effects the photocatalytic degradation efficiency of MXene-based materials. The degradation efficiency decreases as the initial concentration of targeted organic pollutants increases. Various researchers study this effect. For instance, Mollaei *et al.*^114^ examined the photocatalytic degradation of phenol red using its different initial concentrations (*i.e.*, 5, 10, and 20 ppm) using TiO_2_/MXene-2.5 wt%. As shown in Fig. 6e, the % degradation of phenol red decreases from 99.2 to 88.2% as its (phenol red concentration) increases from 5 to 20 ppm after 4 h of irradiation under UV light. This decline in degradation activity can possibly be explained by several factors. First, at higher pollutant concentration the number of organic molecules present in reaction solution increases, which leads to the saturation of active sites available on the MXene-based photocatalyst surface. Resultantly, fewer active sites remain available for photocatalytic reactions, thereby lowering degradation efficiency. Additionally, excessive pollutant molecules can hinder penetration of light and limit the production of electron–hole pairs that are essential for photocatalytic activity. Second, higher pollutant concentrations promote the formation of a larger quantity of intermediate byproducts during degradation process. These intermediates products compete with the parent pollutant molecules for adsorption onto the active sites of MXene-based photocatalyst. Such competition lowers the interaction among the surface or catalyst and the targeted pollutants, ultimately lowering the degradation process. Third, both pollutant molecules and the intermediate products compete for reactive oxygen species (ROS), specifically hydroxyl radicals (˙OH), produced during the photocatalytic process. At higher pollutant concentrations, this competition becomes more intense, lowering the reactive radical availability for effective oxidation of targeted pollutant. Resultantly, the overall degradation efficiency decreases at elevated pollutant concentrations.^137^

### 5.3 Effect of pH

The pH of the reaction solution plays a crucial role in the photocatalytic degradation of toxic and target organic contaminants. This is because pH effectively influences several key factors, such as the surface charge properties of the catalyst, the speciation and dissociation behavior of targeted pollutants, the adsorption ability of the photocatalyst, and the oxidation potential of hydroxyl radicals (˙OH) as well as the valence band (VB) position of the photocatalyst.^138,139^ In MXene-based photocatalytic systems, this pH dependence generally arises through two dominant pathways. The first is oxidant-related, in which the acid dissociation behavior of the applied oxidant, such as persulfate or peroxymonosulfate, determines which reactive radical species predominates at a given pH. The second is surface-charge related, in which the point of zero charge of the photocatalyst relative to the p*K*_a_ of the target pollutant dictates whether electrostatic attraction or repulsion occurs at the catalyst–pollutant interface. The two examples discussed below illustrate each of these pathways in turn. Many studies have been conducted to examine the effect of pH on photocatalytic degradation of organic pollutants using MXene-based materials. For instance, Huang *et al.*^113^ tested the effect of pH on the photocatalytic degradation of SMX using Fe_3_O_4_@MXene composites. SMX can be completely degraded in pH range of 3.0 to 9.0. In this range, the highest and fast degradation of SMX was observed at pH 3.0, while at pH values above 3.0 took much time (Fig. 6f). It was concluded that, acidic medium is more favorable for SMX degradation using Fe_3_O_4_@MXene composites. This may be attributed to several possible reasons: (1) since the p*K*_a_ value of PMS is 9.4,^140^ acidic conditions favors the existence of HSO_5_^−^ species, which facilitates the production of reactive radicals. On the other hand, in alkaline medium, PMS undergoes decomposition to form SO_4_^2−^; (2) in alkaline media, OH^−^ ions can further react with SO_4_˙^–^ to produce ˙OH.^141^ Furthermore, Akbari *et al.*^137^ evaluated the impact of pH on photocatalytic removal of TC using Fe_2_O_3_–SiO_2_/MXene. In this study, highest degradation was examined at pH value of 5.5, whereas lower degradation was observed at highly basic and highly acidic conditions. The point of zero charge (pH_ZPC_) of the Fe_2_O_3_–SiO_2_/MXene material was around pH 6.2, indicating that the surface of catalyst remained positively charged below this pH value (*i.e.*, 6.2). Furthermore, the p*K*_a_ values of TC were 3.3, 7.7, and 9.7, depending on the solution pH. The highest degradation of TC at pH value of 5.5 can be attributed to the electrostatic attraction between positively charged surface of the photocatalyst and negatively charged TC molecules (pH > 3.3). On the other hand, at lower pH values (*i.e.*, <3.3), the catalyst surface and TC molecules both are positively charged which cause repulsion, resultantly slow the degradation process.

### 5.4 Effect of temperature

Temperature is an important operational parameter governing photocatalytic degradation using MXene-based materials; however, its influence is not universally positive and strongly depends on the reaction mechanism and operating conditions. A moderate increase in temperature can accelerate reaction kinetics, enhance molecular diffusion and mass transfer, and promote the activation of oxidants such as PMS in oxidant-assisted photocatalytic systems.^142^ Nevertheless, increasing temperature beyond an optimum level may produce competing effects. Higher thermal energy can favor charge-carrier recombination under certain photocatalytic conditions, thereby decreasing the availability of photogenerated electrons and holes for ROS generation. Elevated temperature may also decrease pollutant adsorption when adsorption is exothermic and can reduce the concentration of dissolved oxygen available for the formation of reactive oxygen species.^143,144^ Therefore, the overall effect of temperature reflects a balance among oxidant activation, reaction kinetics, pollutant adsorption, mass transfer, and photogenerated charge-carrier dynamics rather than a simple monotonic increase in degradation efficiency. Similarly, in Fenton-like system, higher temperature was reported to improve catalytic activity performance by facilitating the activation of oxidants and increasing the reaction kinetics.^42^ Huang *et al.*^113^ studied the effect of temperature on the photocatalytic removal of SMX in the presence of Fe_3_O_4_@MXene, within the range of 20 to 35 °C, which was selected to simulate practical antibiotic-containing wastewater conditions. The obtained results demonstrated that, a slight decrease in degradation of SMX observed at 20 °C which is due to the insufficient activation of PMS at lower temperature.^145^ In contrast, increasing the temperature to 30 and 35 °C slightly enhanced PMS activation, resulting in almost complete (99.9%) SMX degradation (Fig. 6g). In another study, Zhu *et al.*^115^ also studied the removal of methylene blue (MB) at different temperature values using 2D-MX@Fe_3_O_4_. The adsorption experiments showed a notable increase in MB removal efficacy at higher temperature (55 °C). The thermodynamic study confirmed that MB adsorption is feasible and become more favorable as the temperature increases. Specifically, at 55 °C, MB removal was mainly governed by hydrogen bonding combined with electrostatic interactions, whereas at 25 °C, electrostatic interaction driven surface adsorption dominated the activity (Fig. 6h).

## 6. Mechanism of photocatalytic degradation of organic pollutants *via* MXene-based materials

Photocatalytic technology has become an important approach for reducing environmental contamination due to its strong degradation capability. In recent years, various two-dimensional materials have been extensively explored for diverse applications. Among them, MXene, an advanced family of 2D materials first reported in 2011, has attracted significant interest as promising photocatalyst.^34^ MXenes have gained considerable attention because of their remarkable characteristics, such as excellent chemical stability, high surface area, good electrical conductivity, and adjustable surface functionalities, making them highly suitable for different applications.

When photons (*hν*) associated with energy equal to or higher than the bandgap of semiconductor (SC), interact with MXene-based materials, the electrons from the valence band (VB) are transferred to the conduction band (CB) generating electron–hole pairs (e^−^/h^+^) (reaction (1)).^50^ The excited electrons then react with dissolved oxygen (O_2_), resulting in superoxide radicals (O_2_˙^−^) (reaction (2)).^146^ In addition, water molecules take part in the process by reacting with photogenerated holes (h^+^) to produce hydroxyl radicals (reaction (3)).^7^ These radicals (O_2_˙^−^) then interact with protons to produce hydroperoxyl radicals (˙OOH) (reaction (4)).^7^ Thereafter, the two hydroperoxyl radicals combines to form hydrogen peroxide (H_2_O_2_) along with molecular oxygen (O_2_) (reaction (5)).^7^ Hydrogen peroxide can also be prepared through the reduction of oxygen by electrons in the presence of protons (reaction (6)).^147,148^ Subsequently, in the next step electrons generates hydroxyl radicals (˙OH) and hydroxyl ions (OH^−^) by reducing hydrogen peroxide (H_2_O_2_) (reaction (7)),^16^ whereas UV-irradiation of hydrogen peroxide further yields additional hydroxyl radicals (reaction (8)).^149^ Similarly, hydroxide ions also contributes in hydroxyl radical formation by reacting with holes (reaction (9)).^7^ Hydrogen peroxide and superoxide radicals further react to generate oxygen, hydroxide ions, and additional hydroxide radicals (reaction (10)).^149^ Moreover, peroxymonosulfate (PMS) leads to the formation of highly reactive species, including sulfate ions (SO_4_˙^−^) and hydroxyl ions (˙OH) when activated under UV-irradiation (reaction (11)).^150^ PMS can also interact with active radicals such as (SO_4_˙^−^) and (˙OH), resulting in sulfate peroxide radicals (SO_5_˙^−^) through reactions (12) and (13).^113,151^ The two SO_5_˙^−^ when recombined, subsequently yields sulfate radicals and oxygen (reaction (14)).^152^ Similarly, SO_4_˙^−^ and ˙OH radicals may recombine to generate PMS (reaction (15)).^153^ In addition, superoxide radicals (O_2_˙^−^) combine with protons and hydroxyl radicals to generate singlet oxygen (^1^O_2_) through reactions (16) and (17).^113^ Finally, the generated reactive oxygen species, such as O_2_˙^−^, ˙OH, ^1^O_2_, SO_4_˙^−^, and photogenerated holes (h^+^), effectively decompose organic contaminants into less harmful substances such as carbon-dioxide (CO_2_), water (H_2_O), and inorganic ions (reaction (18)). These reaction pathways collectively demonstrate the important role of MXene-based photocatalysts in generating reactive species that efficiently degrade organic pollutants during photocatalytic processes.^37^Fig. 7(a)–(d) represents the mechanism of organic pollutants degradation using MXene-based materials.^45,137,154,155^

**Fig. 7:**
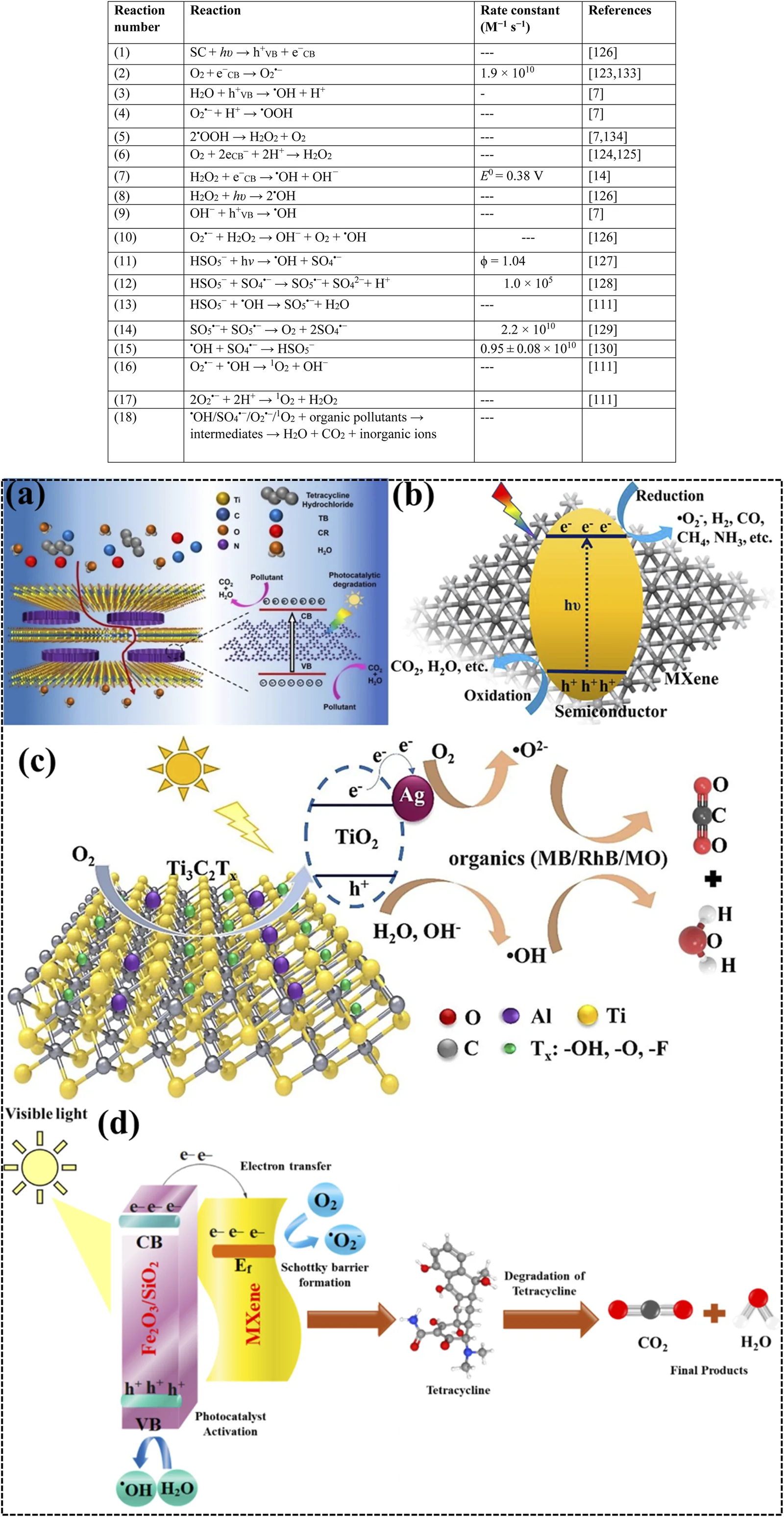
(a) Proposed mechanism of g-C_3_N_4_@MXene/PES composite membrane for the removal of dyes and antibiotics from wastewater. Figure reproduced with permission from ref. 154, Copyright 2022, Elsevier. (b) Generalized mechanism of organic pollutant degradation *via* MXene-based materials. Figure reproduced with permission from ref. 155, Copyright 2021, Elsevier. (c) ROS generation and photodegradation reaction pathways using Ti_3_C_2_T_*x*_/Ti_3_AlC_2_@Ag ternary composite. Figure reproduced from ref. 45, which is open access and permits unrestricted use of materials under the terms of the Creative Commons CC-BY, (d) proposed charge transfer mechanism of the 25FeS/MX-450 photocatalyst under visible-light illumination. Figure reproduced from ref. 137, which is open access and permits unrestricted use of materials under the terms of the Creative Commons CC-BY.

## 7. Kinetics of photocatalytic degradation of organic pollutants using MXene-based materials in the presence of oxidants

MXene-based materials have exhibited remarkable potential for the photocatalytic removal of organic contaminants. In the presence of oxidants (*i.e.*, PS, PMS, and H_2_O_2_), a wide range of contaminants, including pharmaceuticals, pesticides, and dyes, have been effectively removed using MXene-based systems. Various studies were carried out to examine the removal of these organic pollutants from water using MXene-based photocatalytic systems in the presence of oxidant. For example, Zhao *et al.*^123^ fabricated MXene/BiVO_4_/InVO_4_ Z-scheme heterostructure (BIM 30) using hydrothermal approach (Fig. 8a). The surface area of BIM-30 was 45–50 m^2^ g^−1^ (Fig. 8b). The fabricated heterostructure was applied for the photocatalytic degradation of OFX and H_2_O_2_ production. Under the optimized reaction conditions: [catalyst] = 0.267 g L^−1^, [OFX] = 26.53 mg L^−1^, pH = 6.63, 99% of OFX was removed, while TOC reduction of 84.9% was achieved under visible light irradiation in 65.24 min (Fig. 8c). Furthermore, the catalyst showed excellent photocatalytic activity toward the degradation of tetracycline, ciprofloxacin, and cefixime, confirming its good activity. In river, tap, and saline water, BIM 30 obtained degradation efficacy of 93.8, 95.6, and 89.4, respectively. Moreover, Wang *et al.*^116^ fabricated CuS@MXene materials *via* hydrothermal treatment. The BET surface areas of CuS and its composite with MXene were 26.13 and 19.34 m^2^ g^−1^, respectively (Fig. 8d). The as-prepared samples were employed to degrade orange-II (O-II) in the presence of persulfate. The highest degradation of O-II was examined to be 100% using CuS@MXene/PS system in the presence of visible light irradiation, while the % degradation was investigated to be 79, 68, and 62% using PS/vis, CuS@MXene/PS, and CuS@MXene/vis system, respectively, in 60 min (Fig. 8e). In addition, the rate constant (*k*_obs_) value was 0.01615 and 0.02633 min^−1^ for Cus and CuS@MXene, respectively, under visible light irradiation (Fig. 8f). Fig. 8g demonstrate the mechanism of photocatalytic removal of targeted pollutant using CuS@MXene in the presence of PS, while Fig. 8h shows the cycling stability of the composite (*i.e.*, CuS@MXene). Similarly, Iqbal *et al.*^117^ fabricated MXene/CuFe_2_O_4_ composite *via in situ* chemical co-precipitation followed by calcination. The as-prepared material was tested for the photocatalytic removal of ofloxacin (OFL) in the presence of PMS (HSO_5_^−^). Under the reaction conditions of: [MXene/CuFe_2_O_4_] = 500 mg L^−1^, [OFL] = 10 mg L^−1^, [HSO_5_^−^] = 100 mg L^−1^, pH = 6.0, the highest degradation was observed to be 15, 30, 35, 45, 55, 69, 73 and 88% using PMS, MXene/PMS, visible light/PMS, visible light/MXene/PMS, CuFe_2_O_4_/PMS, visible light/CuFe_2_O_4_/PMS, MXene/CuFe_2_O_4_/PMS, and visible light/MXene/CuFe_2_O_4_/PMS systems, respectively. The visible light/MXene/CuFe_2_O_4_/PMS system showed the highest removal efficacy (Fig. 8i). The enhanced photocatalytic activity is attributed to the rapid generation of reactive oxygen species (ROS) under visible light. When as-synthesized material, MXene/CuFe_2_O_4_, is exposed to visible light, photogenerated e^−^ and h^+^ are formed. The e^−^ then react with HSO_5_^−^ to form SO_4_˙^−^ and ˙OH as shown in reaction (19). Fig. 8j illustrates the mechanistic pathways of ofloxacin degradation by SO_4_˙^−^ and ˙OH mediated advance oxidation processes.

19 e^−^ + HSO_5_^−^ → SO_4_˙^−^ + ˙OH

**Fig. 8:**
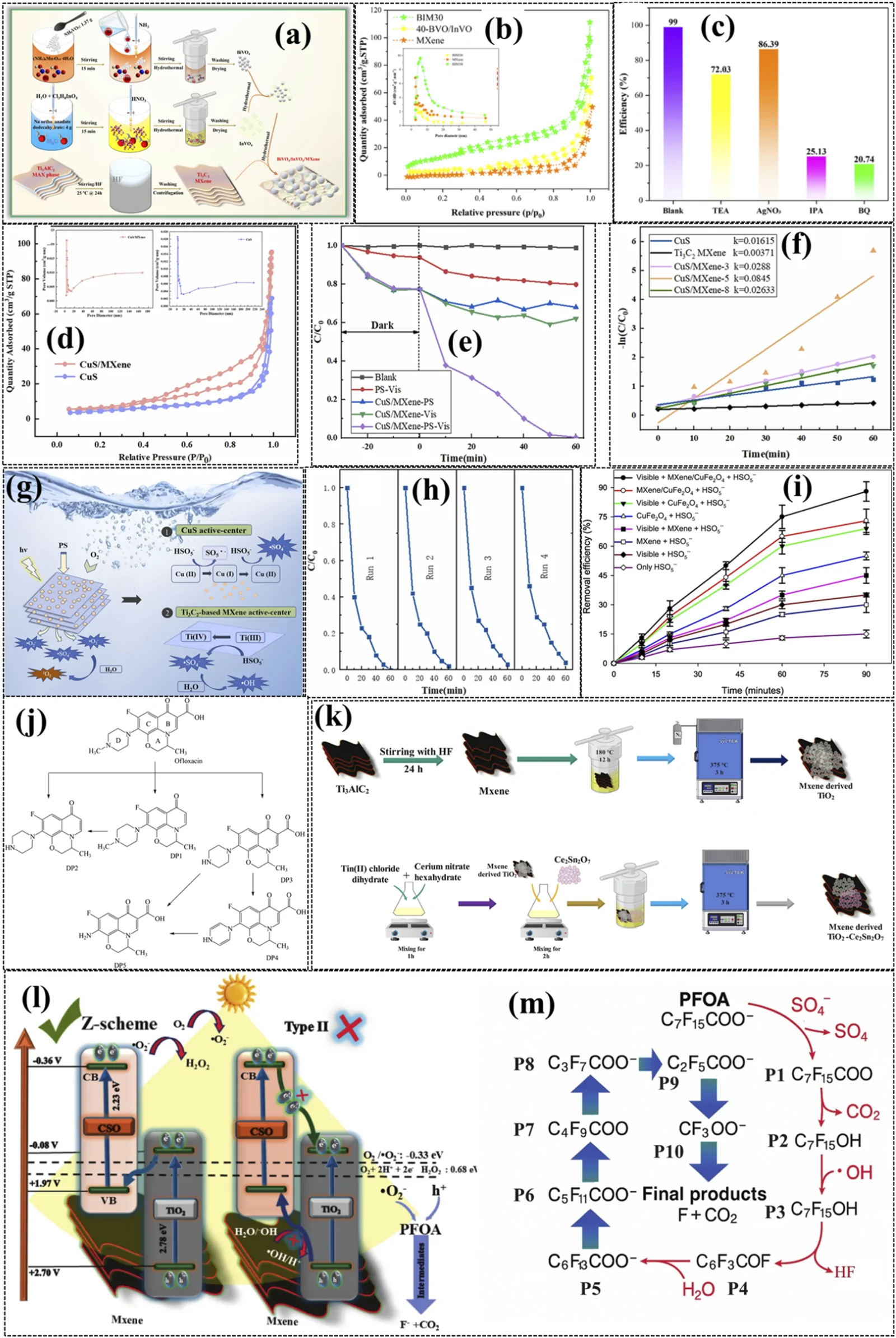
(a) Schematic representation of BIM-30 synthesis process, (b) N_2_ ads–des and BJH pore distribution, (c) scavenger experiments for determination of major reactive species involve in pollutant degradation. Figure reproduced with permission from ref. 123, Copyright 2025, Elsevier (d) N_2_ ads–des isotherms along with pore size distribution profiles (inset) of CuS@MXene and pure CuS. Figure reproduced with permission from ref. 116, Copyright 2022, Elsevier. (e) Degradation efficacy of orange II and (f) corresponding rate constants using CuS, MXene, and CuS@MXene composites with different MXene loadings, (g) mechanism for PS activation using CuS@MXene and (h) recycling stability of the CuS@MXene. Figure reproduced with permission from ref. 116, Copyright 2014, Elsevier (i) removal of OFX using CuFe_2_O_4_, MXene and MXene/CuFe_2_O_4_-activated HSO_5_^−^ systems under dark and visible-light irradiation, (j) proposed OFX degradation pathways involving ˙OH and SO_4_˙^−^. Figure reproduced with permission from ref. 117, Copyright 2025, Elsevier. (k) Synthesis route of MXene-derived TiO_2_ and TiO_2_–Ce_2_Sn_2_O_7_ composites through a HF etching, hydrothermal approach, and calcination, (l) schematic charge-transfer routes pathway showing improved electron–hole separation and radical formation, and (m) proposed mineralization pathway of PFOA into CO_2_ and F^−^. Figure reproduced with permission from ref. 121, Copyright 2026, Elsevier.

Yang *et al.*^121^ prepared MXene-derived TiO_2_–Ce_2_Sn_2_O_7_*via* tow step method, thermal oxidation of MXene to produce titanium dioxide, which followed by coupling of Ce_2_Sn_2_O_7_ through hydrothermal method (Fig. 8k). The surface area and bandgap of as-prepared sample was 232 m^2^ g^−1^ and 2.28 eV, respectively. The as-prepared sample was used for the degradation of PFOA in the presence of H_2_O_2_. The % degradation of PFOA was observed to be 64, 71, 85, 89, and 100% (approximately) using CSO, MXene, MXT-0.2CSO, MXT-0.6CSO, and MXT-0.4CSO in the presence of xenon lamp, respectively, under the experimental conditions of: [catalyst] = 50 mg/100 mL, [PFOA] = 4 mg L^−1^, reaction time = 80 min. Additionally, the *k*_obs_ values were 0.00068, 0.0063, 0.0079, 0.031, and 0.091 min^−1^, accordingly. Fig. 8l shows the mechanism of photocatalytic degradation of PFOA, while Fig. 8m illustrates the stepwise PFOA removal pathways. In conclusion, it was examined that the addition of oxidants (*i.e.*, PS, PMS, and H_2_O_2_) significantly enhances the degradation efficiency of targeted pollutants compared to systems without oxidants.

## 8. Kinetics of photocatalytic degradation of organic pollutants using MXene-based materials in the absence of oxidants

MXene-based materials have also exhibited remarkable potential for the photocatalytic removal of organic contaminants. A wide range of organic pollutants, including pharmaceuticals, pesticides, and dyes, have been effectively removed using MXene-based materials. Different studies were carried out to examine the removal of these organic pollutants from water using MXene-based photocatalytic systems.

For instance, Eslaminejad *et al.*^124^ synthesized palladium nanoparticles anchored on MXene/MOF (Pd/MXOF) nanocomposites through a green synthesis method (Fig. 9a). The synthesized Pd/MXOP nanocomposites showed a surface area of 65.87 m^2^ g^−1^ and a bandgap energy of 1.79 eV (Fig. 9b). In addition, the pore diameter and pore volume of the prepared Pd/MXOF nanocomposite were determined to be 0.0618 cm^3^ g^−1^ and 4.27 nm, respectively. The fabricated nanocomposites were employed as photocatalysts for the degradation of ofloxacin (OFL). Under the experimental conditions of: [Pd/MXOF] = 0.02 g, [OFL] = 20 mg L^−1^, and pH = 5.0, complete degradation of OFL was achieved after 30 minutes of visible light irradiation. To examine the effect of catalyst dosage, several experiments were performed by using various catalyst amounts (0.01, 0.02, 0.03, and 0.04) (Fig. 9c). The degradation efficiency of OFL increased from 55.2% to 100% as the catalyst amount increased from 0.01 to 0.03 g. However, the degradation efficiency decreased to 86.8% at a catalyst dosage of 0.04 g. In this system most of the photoinduced electrons generated in the conduction band of the MOF quickly migrated to MXene and Pd nanoparticle through interfacial electron transfer, resulting in the generation of O_2_˙^−^ radicals. The results indicate that O_2_˙^−^ radicals also play a crucial role in the OFL degradation. Fig. 9d shows the mechanism of photocatalytic removal of OFL using Pd/MXOF nanocomposite. In another study, Cao *et al.*^125^ synthesized the Sn–Bi–MOF/MXene photocatalyst by a solvothermal method. The prepared photocatalyst was utilized for the degradation of tetracycline (TC). Without catalysts, only 9.9% degradation of TC was observed. After 90 min of photocatalytic reaction, the degradation efficiency of Sn–Bi–MOF and MXene were found to be 79.7% and 69.1%, respectively. By the fabrication of MXene into Sn–Bi–MOF/MXene, at pH 11 the degradation of TC increased to 96.2% under xenon lamp irradiation as the light source (Fig. 9e). The synthesized photocatalyst showed surface area of 145.873 m^2^ g^−1^ for Sn–Bi–MOF/MXene and a bandgap energy of 3.02 eV Sn–Bi–MOF. The proposed photocatalytic mechanism of Sn–Bi–MOF/MXene is illustrated in (Fig. 9f). Fig. 9g shows the cycling stability of as-prepared material. Moreover, Furthermore, Aziz *et al.*^126^ synthesized ZnFe_2_O_4_/Cr_2_O_3_/MXene nanocomposite by a hydrothermal method (Fig. 8h). The prepared composite was employed for the photocatalytic degradation of tetracycline (TC). The highest degradation efficiency of TC was recorded as 94.2% under optimized conditions, including a catalyst dosage of 15 mg L^−1^ (Fig. 9i), pollutant concentration of 50 mg L^−1^ and pH 5.0, Xenon lamp irradiation as the light source, and an irradiation period of 1 h. In addition, the calculated rate constant value was 0.0983 min^−1^ (Fig. 9j). The ZnFe_2_O_4_/Cr_2_O_3_/MXene composite shows a bandgap energy level of 2.65 eV and a surface area of 165 m^2^ g^−1^. The proposed mechanism of the photocatalytic degradation of TC using ZnFe_2_O_4_/Cr_2_O_3_/MXene composite as in Fig. 9k.

**Fig. 9:**
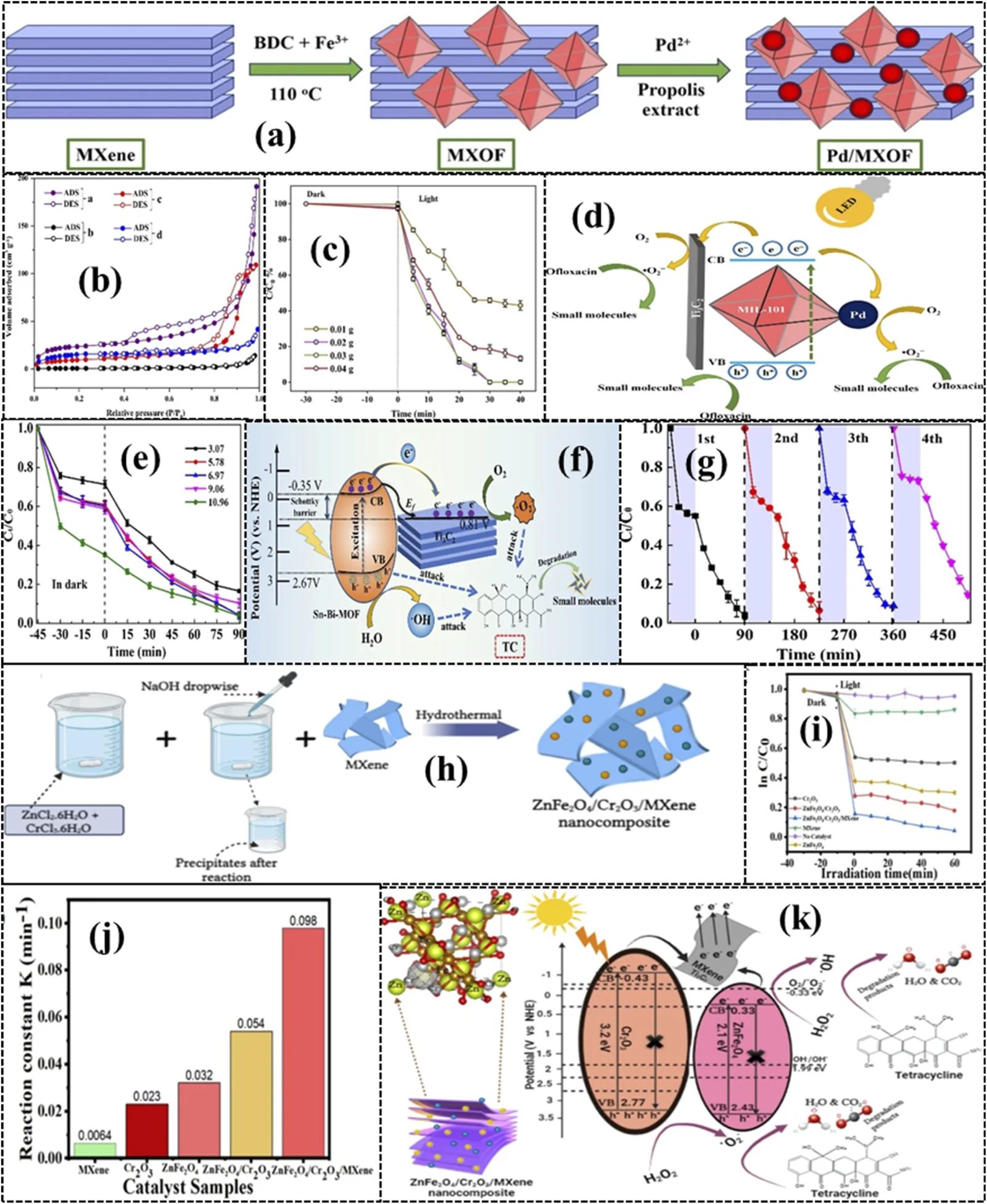
(a) Schematic for the synthesis of Pd/MXOF photocatalyst, (b) N_2_ adsorption–desorption isotherm profiles of MIL-101(Fe), MXOF, MXene, and Pd/MXOF, (c) impact of catalyst dosage on degradation efficacy, (d) possible photocatalytic degradation mechanism of OFL over the Pd/MXOF photocatalyst. Figure reproduced with permission from ref. 124, Copyright 2025, Elsevier (e) influence of pH on photocatalytic removal of TC, (f) suggested photocatalytic reaction mechanism of Sn–Bi-MOF/MXene, and (g) cycling stability of as-prepared sample. Figure reproduced with permission from ref. 125, Copyright 2023, Elsevier (h) synthesis scheme of ZnFe_2_O_4_/Cr_2_O_3_/MXene nanocomposite, (i) photocatalytic removal activity of TC-HCl using Cr_2_O_3_, MXene ZnFe_2_O_4_, ZnFe_2_O_4_/Cr_2_O_3_, and ZnFe_2_O_4_/Cr_2_O_3_/MXene in the presence of visible light, (j) comparative rate constants for as-prepared catalyst, (k) proposed charge separation and charge transfer mechanism involving the formation of O_2_˙^−^ and ˙OH during TC-HCl degradation *via* the ZnFe_2_O_4_/Cr_2_O_3_/MXene nanocomposite. Figure reproduced with permission from ref. 126, Copyright 2025, Elsevier.

In another study, Zairov *et al.*^127^ synthesized a neodymium/dysprosium-based MOF@MXene (Nd/Dy-MOF@MXene) composite through a solvothermal method. The synthesized nanocomposite exhibited a bandgap energy of 2.78 eV (Fig. 10a), while the BET surface area was determined to be 55.21 m^2^ g^−1^. The synthesized nanocomposite was employed for the degradation of methyl orange (MO) dye. Without light the degradation efficiency of MO was found to be 16.23%. However, under light irradiation without a catalyst, the degradation efficiency increased to 27.12%. Moreover, the maximum degradation of MO reached to 93.55% within 30 min under light irradiation in the presence of Nd/Dy-MOF@MXene catalyst (Fig. 10b). Fig. 10c shows the mechanism of MO removal using Nd/Dy-MOF@MXene system. In another study, Claudine *et al.*^128^ synthesized Ti_2_C@GMA/PEDOT: PSS nanocomposites (NCs) using an self-assembly with modification (Fig. 10d). The prepared NCs were applied for the degradation of congo red (CR) dyes and rhodamine B (RhB) (Fig. 10e–h). Under the experimental condition of [Ti_2_C@GMA/PEDOT:PSS] = 10 mg, [RhB] = 10 mg L^−1^, [CR] = 10 mg L^−1^ and reaction time = 60 min, degradation efficiencies of 94.9% and 98.6% were achieved for RhB and CR, respectively, under visible light irradiation. During the photocatalytic process the Ti_2_C MXene substrate played a vital role in promoting efficient charge separation. The incorporation of PEDOT:PSS and glycidyl methacrylate (GMC) enhanced the electrical conductivity and surface activity of the material, thereby improving electron transfer efficiency. The enhanced electron mobility significantly promoted the generation of reactive oxygen species (ROS), such as superoxide radicals (O_2_˙^−^) and hydroxyl radicals (˙OH). These ROS were mainly responsible for the photocatalytic degradation of organic contaminants. Additionally, the calculated rate constant values for RhB and CR degradation were 0.00317 and 0.01044 min^−1^, respectively. To further evaluate the influence of dye concentration on degradation efficiency, a series of experiments were conducted using RhB and CR concentration in range 10 to 50 mg L^−1^. The degradation efficiency of RhB decreased from 94.9% to 92.2% as the RhB concentration increased from 10 to 50 mg L^−1^. Similarly, the degradation efficiency of CR declined from 98.6% to 95.76% with an increase in CR concentration from 10 to 50 mg L^−1^.

**Fig. 10:**
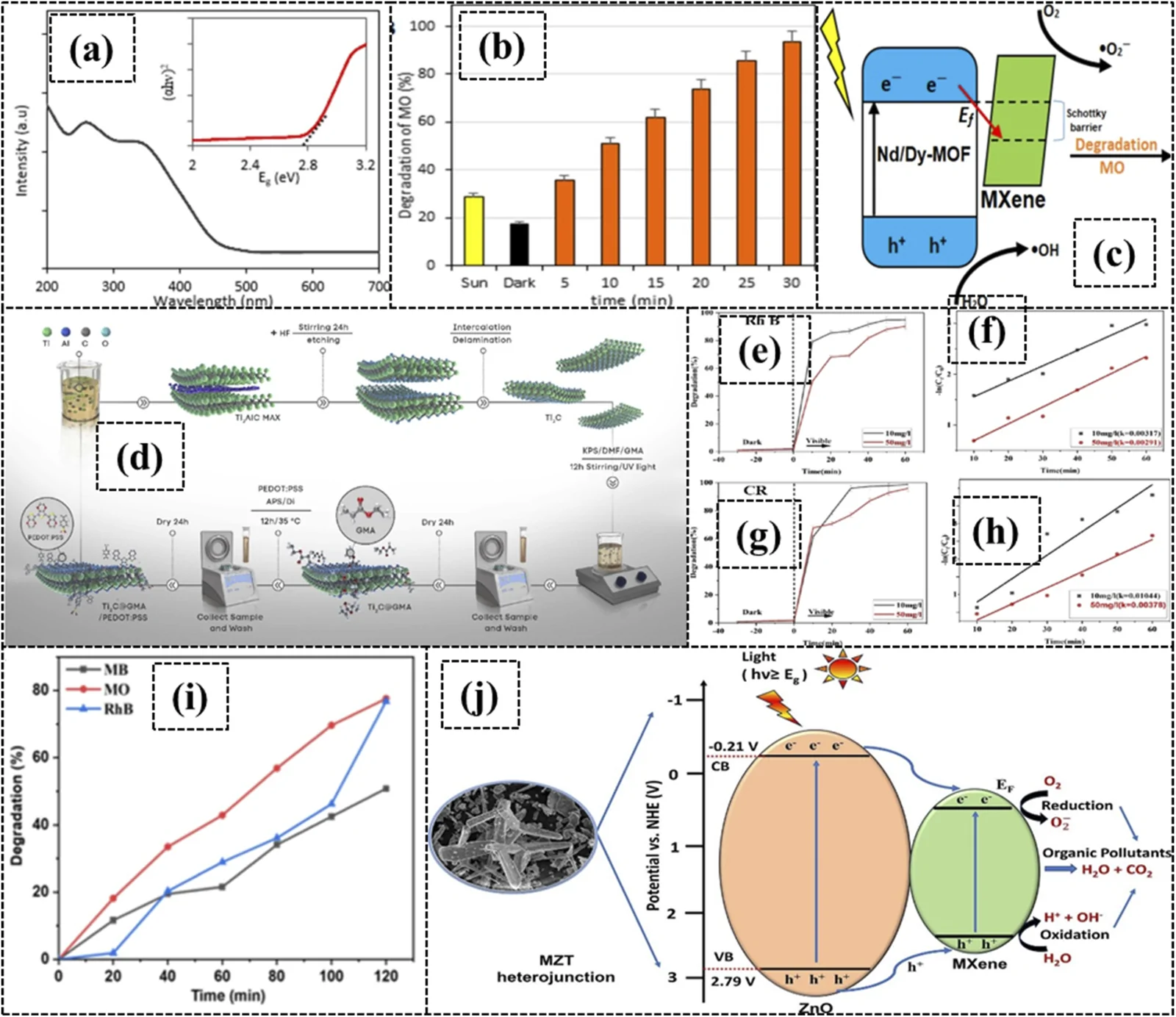
(a) Bandgap analysis of Nd/Dy-MOF@MXene (insert: Tauc plot), (b) percentage degradation observed in various systems, and (c) the proposed mechanism for the photocatalytic degradation of MO. Figure reproduced with permission from ref. 127, Copyright 2025, Elsevier (d) schematic illustration showing the synthesis route of Ti_2_C_3_-based composites, designed to enhance photocatalytic performance through the incorporation of glycidyl methacrylate (GMA), and poly(3,4-ethylenedioxythiophene):poly(styrene sulfonate) (PEDOT:PSS), (e–h) photocatalytic degradation efficiency of the Ti_2_C@GMA/PEDOT:PSS composite for dye removal, (e) photodegradation of rhodamine B (RhB) dye under visible light irradiation, (f) photodegradation of Congo Red (CR) dye under similar conditions, while (g) and (h) represents pseudo-first-order kinetic models describing the photodegradation behavior of RhB and CR at initial dye concentrations of 10 mg L^−1^ and 50 mg L^−1^, respectively, over different reaction times. Figure reproduced with permission from ref. 128, Copyright 2025, Elsevier. (i) shows the time dependent degradation percentage of the dyes, whereas (j) illustrates the proposed mechanism for the photocatalytic activity of MZT. Figure reproduced with permission from ref. 129, Copyright 2014, Elsevier.

Similarly, Jangra *et al.*^129^ synthesized MXene, ZnO-tetrapod (ZT), and MXene decorated ZnO-tetrapod (MZT) nanocomposite. The bandgap energies of MZT, ZT, and MXene were determined to be 2.75, 3.0, and 1.8 eV respectively. In addition, the average pore diameter and surface area of the MZT nanocomposite were found to be 5.82 m^2^ g^−1^ and 22.58 nm, respectively. The synthesized MZT nanocomposite was employed for the photocatalytic degradation of methylene blue (MB), rhodamine B (RhB), and methyl orange (MO) dyes (Fig. 10i). Under the experimental conditions of [MZT] = 50 mg/50 mL, [MB] = [MO] = [RhB] = 10 mg L^−1^, reaction time = 120 min, and sunlight as the light source the maximum degradation efficiencies of MB, MO, and RhB were recorded as 50.83%, 76.79%, and 77.60% respectively. The observed rate constant (*k*_obs_) values for RhB, MO, and MB were 8.9 × 10^−5^, 7.5 × 10^−5^, and 4.1 × 10^−5^ min^−1^ respectively, showing that RhB exhibited a higher *k*_obs_ value compared to MO and MB. This enhanced performance was attributed to the rapid transfer of photogenerated electrons from the valence band of the ZnO to the MXene surface by the interfacial interaction between ZnO and MXene, which effectively suppressed the recombination of charge carriers in the catalyst. Resultantly, under UV-light irradiation, the generated holes (h^+^) directly participated in the degradation of MB, MO, and RhB molecules, while the photogenerated hole in the ZnO valence band remained largely preserved. The superior photocatalytic activity of MZT was basically associated with its low recombination rate, narrow bandgap energy, and due to the presence of oxygen vacancies in the lattice structure, which act as trapping sites for photogenerated electrons hole during photocatalytic process. Fig. 10j demonstrates the mechanism of targeted pollutant degradation using MZT.

## 9. Conclusion and future perspectives

### 9.1 Conclusion

MXene-based photocatalysts have demonstrated considerable potential for the degradation of diverse organic contaminants because their conductive layered structure, tunable surface chemistry, and ability to form intimate interfaces with semiconductor components can facilitate charge separation and interfacial electron transfer. The literature reviewed indicates that photocatalytic performance is strongly influenced by MXene composition, surface termination, morphology, interfacial contact, and the physicochemical properties of the coupled semiconductors. These characteristics determine light utilization, charge-carrier migration, pollutant adsorption, and reactive oxygen species generation. The reviewed studies further demonstrate that photocatalytic degradation is strongly dependent on operating conditions, including photocatalyst dosage, initial pollutant concentration, pH, and reaction temperature. Importantly, degradation efficiency alone does not adequately describe photocatalytic performance. Kinetic analysis provides a more quantitative basis for comparison, and pseudo-first-order behavior is frequently reported for both oxidant-assisted and oxidant-free systems. The observed rate constants demonstrate that appropriate MXene incorporation can accelerate pollutant degradation through improved charge separation and interfacial charge transport. However, direct comparison of reported kinetic parameters remains difficult because experimental conditions vary considerably among studies. Despite the substantial progress demonstrated by the reviewed literature, several research gaps remain. First, systematic comparisons of different MXene compositions and surface terminations under identical experimental conditions are limited, making it difficult to establish clear structure–property–activity relationships. Second, although enhanced charge transfer and suppressed electron–hole recombination are frequently proposed, quantitative correlations between MXene electronic properties, interfacial charge-transfer behavior, reactive species generation, and observed kinetic constants remain insufficient. Third, the identification of dominant reactive species is often based on indirect scavenger experiments, while quantitative determination of ROS contributions and reaction pathways remains limited. Practical implementation also requires further investigation. Many reported studies are conducted using single model pollutants in simplified aqueous systems, whereas real wastewater contains competing organic and inorganic constituents that can alter adsorption, light penetration, ROS generation, and degradation kinetics. In addition, the long-term oxidative stability of MXenes, catalyst recovery and reusability, possible leaching of composite components, and performance over extended operating cycles require more systematic evaluation. Standardized reporting of catalyst dosage, pollutant concentration, irradiation conditions, reactor configuration, pH, and kinetic parameters would also improve meaningful comparison between different MXene-based photocatalysts (Fig. 11).

**Fig. 11:**
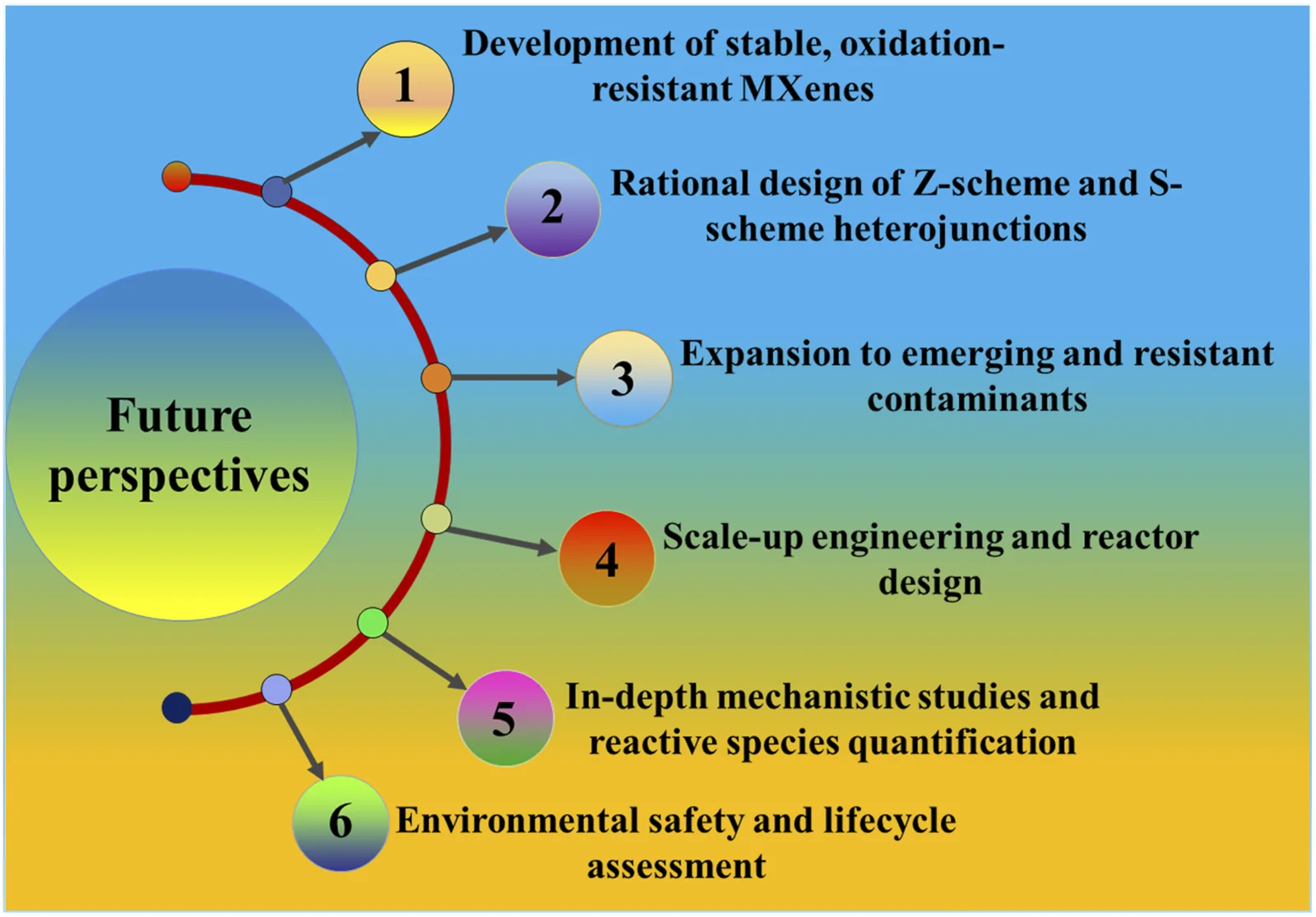
Future perspectives.

### 9.2 Future perspectives

#### 9.2.1 Development of stable, oxidation-resistant MXenes

The most severe problem for the practical application of the MXene-based photocatalysts is their susceptibility to oxidative degradation in water, which gradually transforms the conductive MXene phase to TiO_2_, reducing the catalytic activity. Surface passivation methods such as encapsulation in protective carbon shells, polymer coating, or intentional engineering of stable surface terminal groups should be targeted in the future to enhance the operational cycle of MXene composites. For accurate durability assessment, long-term stability tests will be essential under realistic conditions of wastewater, not in idealized laboratory conditions.

#### 9.2.2 Rational design of Z-scheme and S-scheme heterojunctions

Although MXene composites consisting of binary semiconductor composites are widely studied, the intentional design of the Z-scheme and S-scheme heterojunctions is a promising strategy to efficiently achieve both high utilization of visible light and high oxidative capacity. A next step in the development beyond empirical composite fabrication is computational screening of combinations of MXenes and semiconductors to determine if the interface has optimal charge transfer thermodynamics, which would involve using DFT to pre-screen MXene–semiconductor combinations. This rational design approach could speed up the identification of high-performance systems and decrease the amount of trial and error that currently characterizes the field.

#### 9.2.3 Expansion to emerging and resistant contaminants

Most of the photocatalysis papers published for MXenes have been targeted to a few model dyes and pharmaceuticals under controlled conditions. Considering future investigations, it would be beneficial to expand the scope to cover emerging contaminants of increasing regulatory interest, such as per and polyfluorinated alkane (PFAs) and microplastic-associated pollutants, endocrine disruptors, and antibiotic-resistant genes (ARG) for which the current treatment technologies are not so effective. Testing MXene-based photocatalysts with complex mixtures (multiple pollutants) that represent real industrial or municipal effluents will be an important requirement to ensure their environmental relevance.

#### 9.2.4 Scale-up engineering and reactor design

So far, all the MXene photocatalysis studies have been in idealized batch reactors, and at the milligram to gram scale, under exposure to a single irradiation. An important future challenge is engineering translation of these materials into continuous flow photoreactor configurations, which will be capable of realistic flow rates and pollutant loads. This will involve dealing with practical issues such as catalyst immobilization on supports to prevent separation costs in the reactor downstream, effective light delivery throughout a large reactor volume, and finding scalable synthesis routes of the MXenes that maintain the surface functionality that is essential for photocatalytic applications. Cost-competitive pathways will have to be determined through techno-economic evaluation and lab studies.

#### 9.2.5 In-depth mechanistic studies and reactive species quantification

Although significant advances have been made in their ability to define pollutant degradation to be due to hydroxyl radicals, superoxide anions, and holes, there is a need for more rigorous mechanistic assignments, which are limited using scavenger studies due to the absence of direct spectroscopic quantification of the reactive oxygen species. Advanced *in situ* characterization approaches like electron paramagnetic resonance (EPR), transient absorption spectroscopy, and *operando* X-ray methods are required in future to clearly delineate the pathways for the generation of charge carriers, identification of the active sites, and identification of the reactive species within the system. This mechanistic understanding is important for targeted structural improvement of MXenes to guide their design.

#### 9.2.6 Environmental safety and lifecycle assessment

As photocatalysts approach application, a systematic evaluation of end-of-life, environmental fate and ecotoxicological effects is a critical need. At present, little is known about biosafety, with most information limited to *in vitro* studies. More detailed life cycle assessments (LCA) should be applied in the future, covering the energy consumption of MXene production, the potential release of nanoparticles during photocatalytic use, and toxicity of the surface terminating products under long-term environmental conditions. A comprehensive safety database will be crucial for the regulatory acceptance and responsible commercialization of water treatment technologies based on MXenes.

## Author contributions

N. A.: performed the literature search, formal analysis, and writing – original draft.S. A.: data analysis, and conceptualization, S. K.: formal analysis, and writing – original draft.N. M.: writing – review & editing, and project administration.A. A.: visualization, provided overall supervision.

## Conflicts of interest

The authors declare no competing interests or personal relationship that could be appeared to influence the work in any capacity.

## Data Availability

No primary research results, software or code have been included and no new data were generated or analysed as part of this review.
